# Distinct roles of RAD52 and POLQ in chromosomal break repair and replication stress response

**DOI:** 10.1371/journal.pgen.1008319

**Published:** 2019-08-05

**Authors:** Andrew A. Kelso, Felicia Wednesday Lopezcolorado, Ragini Bhargava, Jeremy M. Stark

**Affiliations:** 1 Department of Cancer Genetics and Epigenetics, Beckman Research Institute of the City of Hope, Duarte, California, United States of America; 2 Irell and Manella Graduate School of Biological Sciences, Beckman Research Institute of the City of Hope, Duarte, California, United States of America; National Cancer Institute, UNITED STATES

## Abstract

Disrupting either the DNA annealing factor RAD52 or the A-family DNA polymerase POLQ can cause synthetic lethality with defects in *BRCA1* and *BRCA2*, which are tumor suppressors important for homology-directed repair of DNA double-strand breaks (DSBs), and protection of stalled replication forks. A likely mechanism of this synthetic lethality is that RAD52 and/or POLQ are important for backup pathways for DSB repair and/or replication stress responses. The features of DSB repair events that require RAD52 vs. POLQ, and whether combined disruption of these factors causes distinct effects on genome maintenance, have been unclear. Using human U2OS cells, we generated a cell line with *POLQ* mutations upstream of the polymerase domain, a *RAD52* knockout cell line, and a line with combined disruption of both genes. We also examined RAD52 and POLQ using RNA-interference. We find that combined disruption of *RAD52* and *POLQ* causes at least additive hypersensitivity to cisplatin, and a synthetic reduction in replication fork restart velocity. We also examined the influence of RAD52 and POLQ on several DSB repair events. We find that RAD52 is particularly important for repair using ≥ 50 nt repeat sequences that flank the DSB, and that also involve removal of non-homologous sequences flanking the repeats. In contrast, POLQ is important for repair events using 6 nt (but not ≥ 18 nt) of flanking repeats that are at the edge of the break, as well as oligonucleotide microhomology-templated (i.e., 12–20 nt) repair events requiring nascent DNA synthesis. Finally, these factors show key distinctions with BRCA2, regarding effects on DSB repair events and response to stalled replication forks. These findings indicate that RAD52 and POLQ have distinct roles in genome maintenance, including for specific features of DSB repair events, such that combined disruption of these factors may be effective for genotoxin sensitization and/or synthetic lethal strategies.

## Author summary

We have examined the role of two factors, RAD52 and POLQ, in genome maintenance pathways. While these factors are biochemically distinct, they are both synthetic lethal with loss of the *BRCA1* and *BRCA2* tumor suppressor genes, and hence are emerging therapeutic targets. Furthermore, RAD52 and POLQ have been implicated in chromosomal break repair events that use flanking repeats to restore the chromosome. We identified distinct features of chromosomal break repair events that are mediated by RAD52 vs. POLQ. Additionally, we have found that combined disruption of *RAD52* and *POLQ* causes at least additive hypersensitivity to cisplatin and a synthetic reduction in replication fork restart velocity. These findings indicate that POLQ and RAD52 have distinct roles in genome maintenance, such that combined disruption of these factors could be a potential therapeutic strategy.

## Introduction

Exploiting synthetic lethal relationships in cancer cells has emerged as a promising therapeutic approach [1, 2]. As a key example, cells deficient in *BRCA1* or *BRCA2* are hypersensitive to inhibitors of Poly-ADP-ribose Polymerase (PARP) [1, 2]. Both *BRCA1* and *BRCA2* are important for homology-directed repair (HDR) of chromosomal breaks, which involves RAD51-mediated invasion of a homologous sequence to template nascent DNA synthesis [3]. In addition, *BRCA1* and *BRCA2* are important for protection of stalled replication forks by blocking recruitment of the MRE11 nuclease to reversed forks [4–6]. PARP inhibitors appear toxic to cells deficient in *BRCA1* and *BRCA2*, by causing DNA lesions that require HDR for repair, and/or replication defects that require protection from degradation via *BRCA1* and *BRCA2* [6]. However, since PARP inhibitors are effective in only a fraction of cancer patients [7], it is important to develop additional targets for this synthetic lethality approach.

In particular, deficiencies in *BRCA1* or *BRCA2* are synthetic lethal with disruption of either RAD52 or POLQ [8–11], which have distinct biochemical activities. RAD52 forms multimeric ring structures and has a strong affinity for ssDNA [12]. Moreover, RAD52 is capable of facilitating the displacement of the ssDNA binding protein replication protein A (RPA) to anneal complementary strands of ssDNA [13, 14]. RAD52 also interacts with dsDNA, although with a weaker affinity than with ssDNA [15]. Consistent with a role in promoting stable DNA annealing, RAD52 appears to protect dsDNA from force-induced strand separation [16]. POLQ is an A-family DNA polymerase that has also been shown to anneal complementary ssDNA [17]. The polymerase domain of POLQ has a unique structure that consists of three insertion loops, which are not conserved among other A-family DNA polymerases [18]. This distinct polymerase domain structure allows for the interaction, annealing, and extension of short ssDNA primers [17, 19]. In addition to its C-terminal polymerase domain, POLQ also has an N-terminal helicase domain [20, 21].

To develop RAD52 and POLQ as therapeutic targets for synthetic lethal approaches, it is important to understand their role in genome maintenance. As one possibility, disruption of *POLQ* or *RAD52* may cause similar effects as PARP inhibitors, e.g., cause defects at replication forks that require *BRCA1* and *BRCA2* [6]. Although, an additional potential mechanism of such synthetic lethality is that these factors mediate alternative DSB repair pathways to HDR. In particular, one class of repair pathways involves annealing of homologous repeat sequences that flank the break. These pathways are referred to as Single Strand Annealing (SSA) and Alternative end-joining (Alt-EJ), which generally are distinguished by the use of long vs. short repeat sequences (the latter referred to as microhomology), and the involvement of RAD52 vs. POLQ, respectively [22–28]. However, a limitation of these terms is that the precise parameters that define the mechanism of these events remain poorly understood. Such parameters include repeat length, and influence of a non-homologous intervening sequence. Thus, we refer to these events collectively as repeat-mediated repair (RMR) to avoid a presumption of mechanism.

In this study, we have sought to define whether RAD52 and POLQ have distinct vs. redundant functions in chromosomal break repair or response to replication stress. Specifically, we have examined the influence of these factors on several distinct features of DSB repair, as well as in response to genotoxic agents and replication stress. To test whether these factors have distinct (i.e., non-epistatic) roles in these aspects of genome maintenance, we have also compared cells with combined deficiency in POLQ and RAD52 vs. cells with disruption of the individual factors. Finally, we posited that RAD52 and POLQ have distinct roles in genome maintenance vs. BRCA2, due to their synthetic lethality with BRCA2 loss. Thus, we have also compared the influence of these factors vs. BRCA2 on DSB repair events and in response to replication stress.

## Results

### Combined deficiency of RAD52 and POLQ causes at least additive hypersensitivity to cisplatin

We have sought to examine the relative roles of RAD52 and POLQ in cellular response to genotoxic stress, including distinct DSB repair events. For this, we developed cell lines with disruptions of these genes (both single and double mutants) using the RNA-guided nuclease Cas9. For our parental cell line, we used human osteosarcoma U2OS cells [29, 30], which retain intact cell cycle checkpoints [31, 32]. Notably, these cells rely on the ALT-pathway of telomere maintenance, which could possibly influence repair mechanisms [33]. Our parental cell line was also stably transfected with pFRT/lacZeo (i.e., U2OS Flp-In T-Rex) [29, 30], which is used to integrate the reporter assays described below.

**Fig 1:**
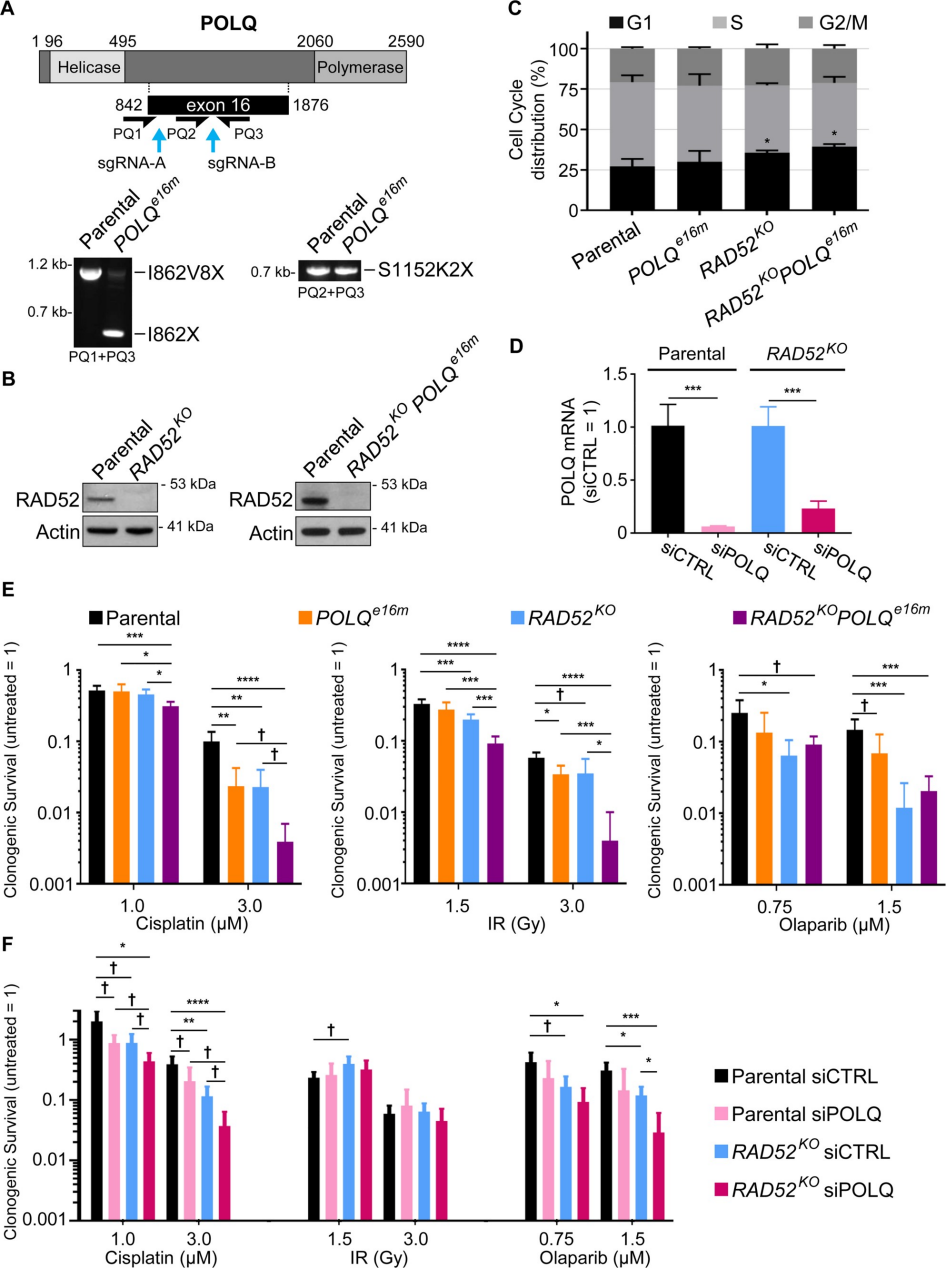
Combined disruption of RAD52 and POLQ causes at least additive sensitivity to cisplatin. (A) Generation of the *POLQ*^*e16m*^ cell line from the parental line (U2OS Flp-In T-REx). Shown is a diagram of POLQ along with two sgRNAs (A and B) used to target Cas9 to exon 16 of *POLQ*. Shown are amplification products of the Cas9 target sites using POLQ exon 16 primers (PQ1, PQ2, and PQ3), which were analyzed by sequencing to identify three mutant alleles: I862X (loss of fragment), I862V8X (inversion of fragment), and S1152K2X (1 nt insertion at the sgRNA-B cut site). (B)The *RAD52*^*KO*^ and *RAD52*^*KO*^*POLQ*^*e16m*^ cell lines were generated by Cas9 using sgRNAs targeting RAD52, and isolated by screening clones using RAD52 immunoblot analysis. Shown are RAD52 immunoblot signals from these cell lines and the parental line, with Actin as a loading control. (C)Cell cycle phases (G1, S, G2/M) for each cell line, based on BrdU labeling and propidium iodide counterstain. Error bars represent SD. *n* = 5 for parental, *n* = 4 for *POLQ*^*e16m*^, and *n* = 3 for *RAD52*^*KO*^ and *RAD52*^*KO*^*POLQ*^*e16m*^. * *P* < 0.05, comparison of percentage in G1 vs. parental, using unpaired *t*-test with Holm-Sidak correction. (D) Shown is POLQ mRNA abundance in parental and *RAD52*^*KO*^ cells treated with a pool of four siRNAs targeting POLQ (siPOLQ) or non-targeting siRNA (siCTRL). POLQ mRNA abundance is based on threshold cycle (Ct) values from PCR amplification, normalized to actin, and relative to siCTRL treated cells (siCTRL = 1). Error bars represent SD. *n* = 3 PCR amplifications, and *** *P* < 0.005, using unpaired *t*-test with Holm-Sidak correction. (E) Shown is fraction clonogenic survival for each cell line treated with cisplatin, ionizing radiation (IR), or Olaparib, each normalized to untreated (untreated = 1). Error bars represent SD, and *n* = 6. * *P* < 0.05, ** *P* < 0.01, *** *P* < 0.005, **** *P* < 0.001, using unpaired *t*-test with Holm-Sidak correction, † *P* < 0.05 using unpaired *t*-test, but not significant when corrected for multiple comparisons (i.e., unadjusted *P*-value). (F) Shown is the fraction clonogenic survival for parental and *RAD52*^*KO*^ cells treated with siCTRL or siPOLQ, exposed to cisplatin, IR, or Olaparib, each normalized to untreated (untreated = 1). Error bars represent SD, and *n* = 6. † *P* < 0.05 (unadjusted *P*-value), * *P* < 0.05, ** *P* < 0.01, *** *P* < 0.005, **** *P* < 0.001, using unpaired *t*-test with Holm-Sidak correction.

We used single guide RNAs (sgRNAs) and Cas9 to generate cell lines deficient in POLQ and RAD52. To generate a POLQ-deficient cell line, we used two sgRNAs targeting exon 16 (Fig 1A). We targeted this region of *POLQ* to disrupt expression of the C-terminal polymerase domain, and thereby cause loss of POLQ-mediated primer extension [20]. We screened for clones with deletion mutations by PCR, and identified a clone with three mutations in exon 16 (*POLQ* *e*xon *16 m*utant, *POLQ*^*e16m*^): 1) one allele with deletion of the segment between the two DSBs, causing mutation of I862 to a termination codon (I862X), 2) a second allele with an inversion of this segment causing mutation of I862 to V, and encoding another 8 amino acids followed by a termination codon (I862V8X), and 3) a third allele with a single nucleotide insertion at the 3' DSB site causing an S1152 to K mutation, and encoding 2 amino acids followed by a termination codon (S1152K2X) (Fig 1A). These mutant alleles disrupt the coding sequence for *POLQ* upstream of the C-terminal polymerase domain (Fig 1A). We also used Cas9 to generate a *RAD52* knockout (*RAD52*^*KO*^) cell line, and a *RAD52*^*KO*^*POLQ*^*e16m*^ cell line from the *POLQ*^*e16m*^ cell line, both of which were identified using RAD52 immunoblotting (Fig 1B). Using these cell lines, we first examined cell cycle profiles using BrdU and propidium iodide labeling, and found that *RAD52*^*KO*^ and *RAD52*^*KO*^*POLQ*^*e16m*^ cells, but not *POLQ*^*e16m*^ cells, showed a modest, but statistically significant increase in G1 cells compared to the parental cell line (Fig 1C).

To examine the response to genotoxic stress, we exposed cells to DNA damaging agents and measured clonogenic survival based on colony formation. In addition to testing the cell lines described above, we also examined POLQ using RNA-interference (RNAi). Specifically, we treated parental and *RAD52*^*KO*^ cells with siRNAs targeting POLQ (siPOLQ), or a non-targeting siRNA (siCTRL). We confirmed that siPOLQ treatment causes depletion of the POLQ mRNA in both the parental and *RAD52*^*KO*^ cells (Fig 1D). Beginning with the crosslinking agent cisplatin, we examined the effect of two doses of cisplatin on clonogenic survival. At the higher dose, we found that both the *RAD52*^*KO*^ and *POLQ*^*e16m*^ cell lines were hypersensitive, compared to the parental cell line (Fig 1E). Furthermore, the *RAD52*^*KO*^*POLQ*^*e16m*^ cells were hypersensitive compared to both the parental cells and the single mutants, at both doses (Fig 1E). Notably, the fold-effect on clonogenic survival for the *RAD52*^*KO*^*POLQ*^*e16m*^ cells was at least additive, compared to the effects of the single mutants (Fig 1E). Similarly, we found that siPOLQ treatment caused hypersensitivity to cisplatin at both doses, in both the parental and *RAD52*^*KO*^ cells (Fig 1F). Finally, the *RAD52*^*KO*^ cells treated with siPOLQ showed at least additive hypersensitivity to cisplatin, as compared to the effects of siPOLQ treatment in the parental cell line, and the *RAD52*^*KO*^ cells vs. the parental cells (Fig 1F). Thus, disruption of RAD52 and POLQ appear to cause hypersensitivity to cisplatin, which is at least additive with combined disruption of these factors.

We also examined clonogenic survival in response to ionizing radiation (IR), and the PARP inhibitor Olaparib. Using two doses of IR, we found that the single mutant cell lines either showed no hypersensitivity, or showed a modest hypersensitivity (Fig 1E, < 2-fold). Similarly, siPOLQ treatment did not caused an obvious effect on IR response in either the parental or *RAD52*^*KO*^ cells (Fig 1F). In contrast, the *RAD52*^*KO*^*POLQ*^*e16m*^ cells showed significant hypersensitivity to both doses of IR (Fig 1E). These findings indicate that RAD52 and POLQ have modest effects on resistance to IR. Although, the results from the *RAD52*^*KO*^*POLQ*^*e16m*^ cell line indicate that combined genetic disruption of these factors can cause IR hypersensitivity. Using two doses of Olaparib, the *RAD52*^*KO*^ and *RAD52*^*KO*^*POLQ*^*e16m*^ cells were both hypersensitive compared to the parental cell line at both doses (Fig 1E). The *RAD52*^*KO*^ and *RAD52*^*KO*^*POLQ*^*e16m*^ cells were not statistically different from each other (Fig 1E). The *POLQ*^*e16m*^ and siPOLQ-treated parental cells showed a modest hypersensitivity to Olaparib (≤ 2.1-fold), and siPOLQ-treatment in the *RAD52*^*KO*^ cell line caused hypersensitivity to Olaparib at both doses (Fig 1F). Thus, RAD52 and POLQ appear important for resistance to Olaparib, although RAD52 appears to have a greater effect.

### RAD52 and POLQ promote RMR events with distinct repeat lengths

**Fig 2:**
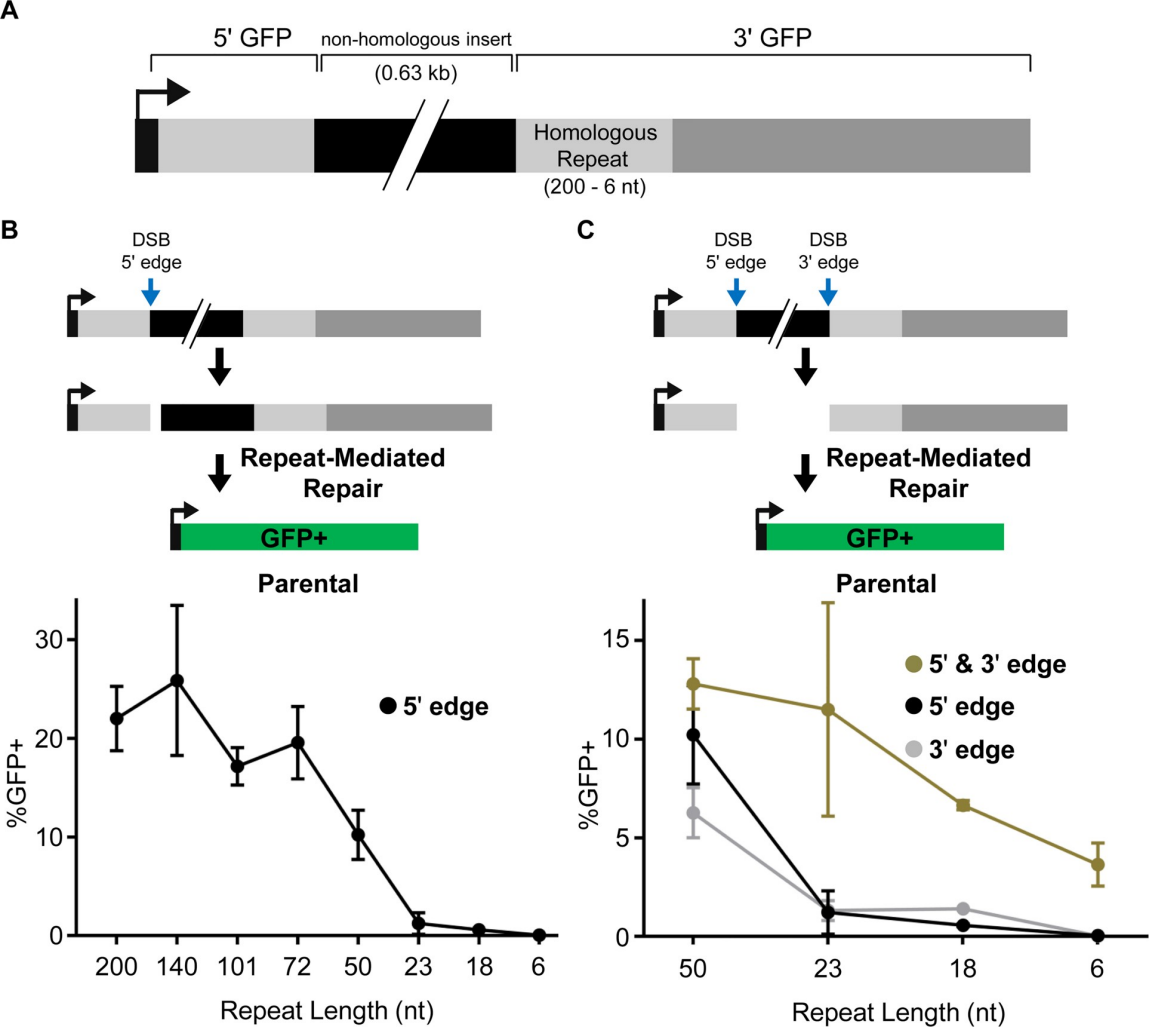
RMR frequencies in parental U2OS cells. (A) Repeat-mediated repair (RMR) reporter diagram. The GFP coding sequence is disrupted by a non-homologous insert, and for each reporter the 3' GFP fragment shares homology with the 5' GFP fragment, but with varying lengths of homology (200–6 nt). Each reporter was integrated into cells using the FRT/Flp system. (B) GFP+ repair events induced by an sgRNA/Cas9-mediated DSB targeted to the 5' edge of the non-homologous insert (5' edge). Shown is the GFP+ frequency induced by the 5' edge DSB for each reporter cell line with varying lengths of homology in the 3' GFP fragment (200–6 nt), each normalized to transfection efficiency. Two independent clones were tested for each reporter with four independent replicates, for a total of *n* = 8, except for the 23 nt reporter where six independent clones were tested for a total of *n* = 24. Error bars represent SD. (C) GFP+ repair events induced by expressing sgRNAs with Cas9 that cause DSBs at the 5' edge of the non-homologous insert (5' edge), the 3' edge of the non-homologous insert (3' edge), or both the 5' and 3' edge of the non-homologous insert (5' & 3' edge). Shown is the percentage of GFP+ cells for each reporter cell line with ≤ 50 nt of homology in the 3' GFP fragment (50–6 nt), which was normalized to transfection efficiency. Error bars represent SD. The number of clones tested and total *n* as in (B).

We then sought to examine the influence of RAD52 and POLQ on distinct DSB repair events. Both RAD52 and POLQ have been implicated in DSB repair that uses homologous repeat sequences that flank a DSB to bridge the break [23, 34]. These events often cause a deletion between the repeat, along with one copy of the repeat, such that we refer to all of these events as repeat-mediated repair (RMR). The parameters of RMR events that are mediated by RAD52 vs. POLQ have remained unclear. Thus, we sought to establish a reporter assay platform to examine two variable features of RMR events: repeat length and non-homologous tail removal. For this, we generated a set of reporter assays in which an expression cassette for green fluorescent protein (GFP) was disrupted by a non-homologous insert sequence (Fig 2A). We then added a homologous repeat of varying lengths (200–6 nt), by expanding the size of the 3' GFP sequence (S1A Fig). Each reporter was integrated in the U2OS cell lines, using the FRT/Flp system [35] (S1B and S1C Fig). In these reporter assays, the RMR events are induced by expression of Cas9 and various sgRNAs.

We tested these reporters in the parental U2OS cells with different combinations of DSBs. To begin with, we targeted a DSB at the 5' edge of the non-homologous insert, such that an RMR event that uses the flanking homology would restore the GFP expression cassette (Fig 2B). In the parental cells, we found that inducing this DSB in the reporters with repeat lengths of 200–72 nt caused similar frequencies of GFP+ cells (Fig 2B). However, with a repeat length of 50 nt, the frequency of GFP+ events was reduced approximately 2-fold compared to the longer repeats, and with repeat lengths of 23–6 nt, induction of GFP+ cells was nearly abolished (Fig 2B). We then considered that the inability to detect RMR events at the shorter repeats was due to the presence of the non-homologous insert. So, we examined these reporters using two DSBs to excise the insert: the first sgRNA targets the edge of the 5' GFP sequence as described above, and the second sgRNA targets the edge of the 3' GFP sequence, which is distinct for each reporter (5' & 3' edge; Fig 2C). With this approach, we were able to readily detect GFP+ events at each of the shorter repeat lengths (50–6 nt, Fig 2C). Notably, as with the 5' edge DSB alone, the 3' edge DSB alone was insufficient to significantly induce GFP+ cells for repeat lengths of 23–6 nt (Fig 2C). Thus, both DSBs are required to significantly induce these repair events. The restoration of the GFP coding sequence was confirmed for each of the reporters by sorting cells to enrich for GFP+ cells, followed by PCR amplification and sequencing analysis (S2A Fig).

**Fig 3:**
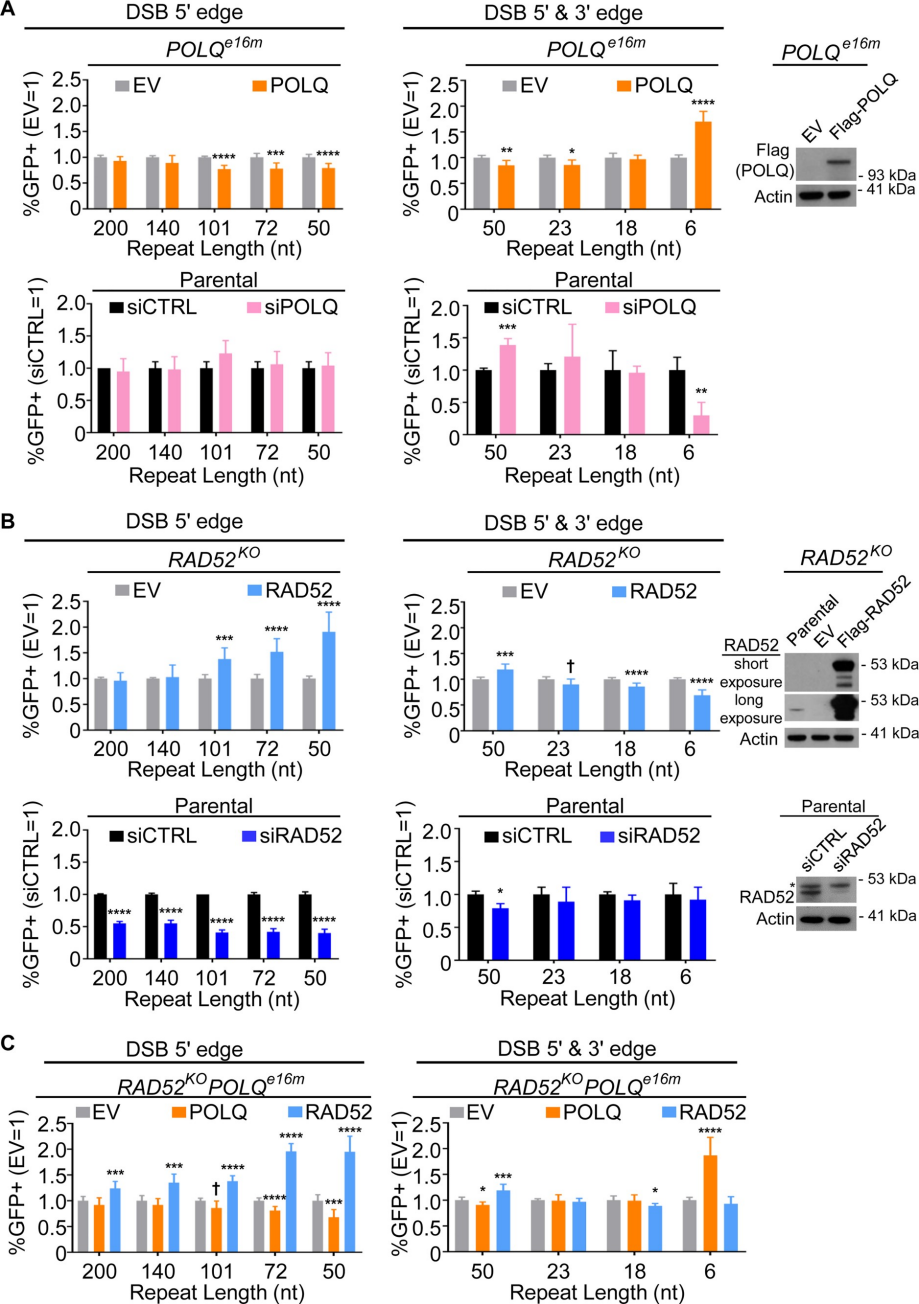
RAD52 and POLQ promote distinct RMR events. (A) Influence of POLQ on RMR events induced by sgRNA/Cas9-mediated DSBs targeted to the 5' edge of the non-homologous insert (5' edge) or to both the 5' edge and 3' edge of the non-homologous insert (5' & 3' edge), as shown in Fig 2. The *POLQ*^*e16m*^ reporter cell line was transfected with an expression vector for the sgRNA(s) and Cas9, as indicated, along with empty vector (EV) or POLQ expression vector. GFP+ frequencies are normalized both to transfection efficiency and to parallel EV samples (EV = 1). Two independent clones were tested for each reporter in each cell line with four independent replicates, for a total *n* = 8. Also shown is immunoblot analysis confirming expression of POLQ (Flag immunotag) from the complementation vector, with Actin as a loading control. For the siRNA treatment, parental cells with each reporter were pre-treated with the respective siRNA, and then co-transfected with the sgRNA(s) and Cas9 expression plasmid shown, along with either non-targeting siRNA (siCTRL) or a pool of four POLQ siRNAs (siPOLQ). GFP+ frequencies are normalized to transfection efficiency and shown relative to the non-targeting siRNA (siCTRL = 1). Each reporter was tested twice with two independent replicates for a total *n* = 4. Error bars represent SD. † *P* < 0.05 (unadjusted *P*-value), * *P* < 0.05, ** *P* < 0.01, *** *P* < 0.005, **** *P* < 0.001, EV vs. complementation, and siCTRL vs. siRNA treatment using unpaired *t*-test with Holm-Sidak correction. (B) Influence of RAD52 on RMR events induced by sgRNA/Cas9-mediated DSBs targeted to the 5' edge or 5' & 3' edge, as in (A). The *RAD52*^*KO*^ reporter cell line was transfected with an expression vector for the sgRNA(s) and Cas9, as indicated, along with EV or RAD52 expression vector. GFP+ frequencies are normalized both to transfection efficiency and to parallel EV samples (EV = 1). Shown is immunoblot analysis confirming expression of RAD52 from the complementation vector in the *RAD52*^*KO*^ cell line. A long exposure blot was included to show endogenous RAD52 expression in the parental line. For siRNA treatment of the parental cells, each reporter was co-transfected with the sgRNA(s) and Cas9 expression plasmid shown, along with siCTRL or a pool of four RAD52 siRNAs (siRAD52). Immunoblot analysis shows the depletion of RAD52 with siRNA in the parental line. Actin was a loading control. Frequencies of GFP+ cells analyzed, number of cell lines tested, number of replicates, and statistics are as in (A). (C) Influence of RAD52 and POLQ on RMR events induced by sgRNA/Cas9-mediated DSBs targeted to the 5' edge or 5' & 3' edge, as in (A) but in the *RAD52*^*KO*^*POLQ*^*e16m*^ cell line. The *RAD52*^*KO*^*POLQ*^*e16m*^ cell line was transfected with an expression vector for the sgRNA(s) and Cas9, as indicated, along with EV, POLQ expression vector, or RAD52 expression vector. GFP+ frequencies are normalized both to transfection efficiency and to parallel EV samples (EV = 1). Frequencies of GFP+ cells analyzed, number of cell lines tested, number of replicates, and statistics are as in (A).

We then analyzed the influence of RAD52 and POLQ on this series of RMR events. Since the *RAD52*^*KO*^, *POLQ*^*e16m*^, and *RAD52*^*KO*^*POLQ*^*e16m*^ cell lines were generated using the U2OS Flp-In T-REx cell line, we were able to integrate each reporter into these lines using the FRT/Flp system. At least two independent integrants of each reporter for each cell line were analyzed. A technical limitation of DSB reporter assay experiments is that different cell lines and experimental replicates can show variations in transfection efficiency, although this issue is partially mitigated by normalizing each experiment to transfection frequency using a parallel well with a GFP expression vector. To address this technical limitation via another method, we used transient complementation, which enables examination of the same cell line with parallel transfections of the complementation vector vs. empty vector (EV). The complementation vector was included in the transient transfection with the sgRNA/Cas9 plasmid(s). However, a drawback of this approach is that complementation vectors do not readily mimic endogenous levels of the respective protein. Indeed, for the POLQ complementation vector, while we confirmed expression using the Flag-immunotag (Fig 3A), we were unable to identify an antibody that is sensitive to detect endogenous POLQ. Thus, we were unable to compare endogenous POLQ levels vs. expression from the complementation vector. Furthermore, while we used a relatively low concentration of the RAD52 complementation vector, we found that these experimental conditions caused a marked increase in RAD52 protein levels (Fig 3B).

Accordingly, in addition to using complementation analysis, we also independently assessed the influence of RAD52 and POLQ on these RMR events using RNAi, by treating cells with siRNAs targeting these factors (siRAD52 and siPOLQ, respectively). As with complementation experiments, RNAi enables comparisons of the same cell line with parallel transfections (i.e., the targeting siRNA vs. siCTRL). As mentioned above, we confirmed that siPOLQ treatment causes depletion of the POLQ mRNA in these cell lines (Fig 1D). We also confirmed that siRAD52 causes a reduction in RAD52 protein (Fig 3B).

Beginning with POLQ, we found several repair events were reduced in *POLQ*^*e16m*^ cells compared to the parental line (S3A Fig). However, POLQ expression in the *POLQ*^*e16m*^ cells promoted only one RMR event: the RMR with the 6 nt repeat, which was induced using two DSBs to excise the non-homologous insert (5' & 3' edge; Fig 3A). Similarly, siPOLQ caused a significant decrease in the 6 nt repeat RMR event, but not any of the others (i.e., RMR events with ≥ 18 nt repeats) (Fig 3A).

For RAD52, beginning with the RMR events with repeat lengths of 200–50 nt and using the 5' edge DSB, the *RAD52*^*KO*^ cell line exhibited lower frequencies vs. the parent cell line for each of these events (S3B Fig). Regarding complementation, we found that RAD52 expression in the *RAD52*^*KO*^ cells significantly promoted these events for repeat lengths of 101, 72, and 50 nt, but not for repeats of 200 and 140 nt (Fig 3B). We found similar results with the *RAD52*^*KO*^*POLQ*^*e16m*^ cell line, although in this case RAD52 expression promoted each of these repair events (i.e., 200–50 nt and using the 5' edge DSB, Fig 3C, S3C Fig). Similarly, siRAD52 treatment caused a significant reduction in each of these repair events (200–50 nt and using the 5' edge DSB, Fig 3B).

We then examined the influence of RAD52 on the events with shorter repeats (50–6 nt) by inducing two DSBs to excise the non-homologous insert (i.e., 5' & 3' edge). The *RAD52*^*KO*^ and *RAD52*^*KO*^*POLQ*^*e16m*^ cells showed reduced frequencies of several of these events, compared to the parental line (S3B and S3C Fig). However, we found that RAD52 expression in *RAD52*^*KO*^ and *RAD52*^*KO*^*POLQ*^*e16m*^ cells showed only a modest increase in events for the 50 nt repeat (1.2-fold), and did not promote events involving 23, 18, or 6 nt (Fig 3B and 3C). Similarly, again using the two DSBs to excise the insert, siRAD52 treatment caused a modest reduction in the event with the 50 nt repeat, but did not affect the frequencies of the events with the shorter repeat lengths (Fig 3B).

Altogether, considering effects of both complementation and RNAi, these findings indicate that RAD52 is important for RMR events using ≥ 50 nt repeats, whereas POLQ promotes RMR events using 6 nt repeats, but not ≥ 18 nt. Regarding the double-mutant cell line, as mentioned above, the findings were similar for the *RAD52*^*KO*^ and *RAD52*^*KO*^*POLQ*^*e16m*^ cell lines with this panel of reporters and complementation analysis (Fig 3C). We also found that POLQ complementation in the *RAD52*^*KO*^*POLQ*^*e16m*^ cell line showed the same results as with the *POLQ*^*e16m*^ cell line (i.e., promoted only the RMR event using the 6 nt repeat; Fig 3C). Thus, the analysis with the *RAD52*^*KO*^*POLQ*^*e16m*^ cell line indicates that combined disruption of *RAD52* and *POLQ* does not appear to generate a synthetic defect in RMR events, but rather shows a combination of two independent defects found in the single mutant cell lines.

### RAD52 is important for RMR events that require removal of a non-homologous sequence

**Fig 4:**
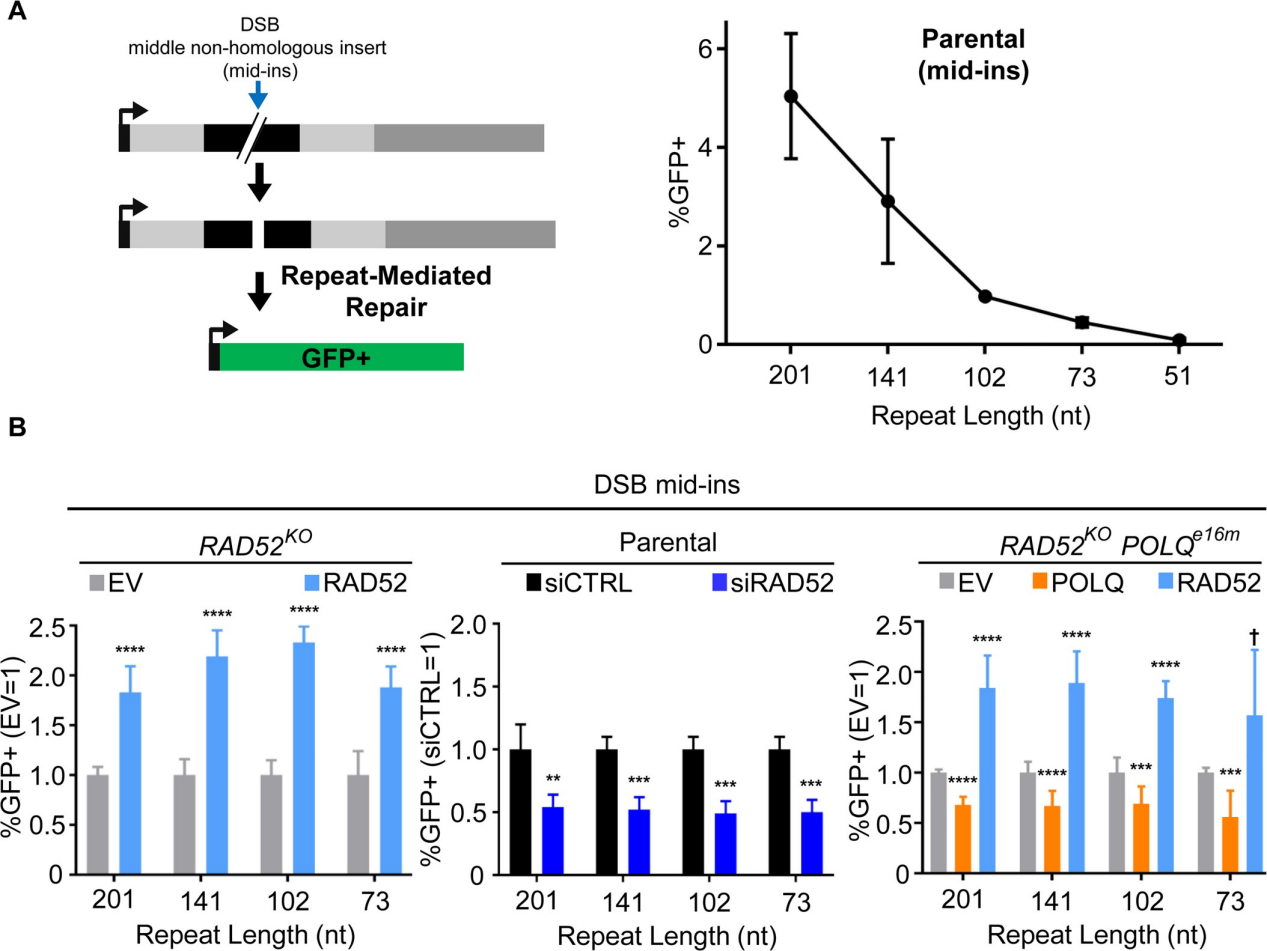
RAD52 is important for RMR events that require removal of a non-homologous sequence. (A) GFP+ repair events induced by an sgRNA/Cas9-mediated DSB targeted to approximately the middle of the non-homologous insert (mid-ins). Frequencies of GFP+ repair events in parental cells are shown normalized to transfection efficiency. Error bars represent SD, and two independent clones were tested for each reporter with four independent replicates for a total *n* = 8. (B) Influence of RAD52 on RMR events induced by a DSB in the mid-ins position. *RAD52*^*KO*^ and *RAD52*^*KO*^*POLQ*^*e16m*^ reporter cell lines were transfected with a plasmid expressing the mid-ins sgRNA and Cas9, along with EV, RAD52 expression vector, and for the latter cell line, the POLQ expression vector. GFP+ frequencies are normalized to transfection efficiency and shown relative to EV (EV = 1). Two independent clones were tested for each reporter in each cell line with four independent replicates for a total *n* = 8. Experiments with siRNA were performed as in Fig 3. Each reporter was tested twice with two independent replicates for a total *n* = 4. Error bars represent SD. † *P* < 0.05 (unadjusted P-value), ** *P* < 0.01, *** *P* < 0.005, **** *P* < 0.001, EV vs. complementation, and siCTRL vs. siRAD52, using unpaired *t*-test with Holm-Sidak correction.

In the above analysis, for RMR events with a 50 nt repeat, we found that RAD52 is more important for such events when induced by one DSB at the 5' edge, compared to when the non-homologous sequence was excised with the 5' & 3' edge DSBs (Fig 3B). The distinction between these events is that the former requires the removal of the non-homologous sequence upstream of the 3' GFP segment. Accordingly, we sought to also examine RMR events requiring removal of non-homologous sequences from both sides of the DSB. To test this, we used an sgRNA to induce a DSB approximately in the middle of the non-homologous insert (mid-ins; 0.3 kb from both 5' GFP and 3' GFP). Using this mid-ins DSB, the repeat lengths are increased by 1 nt, compared to the above analysis (Fig 4A), since the 5' DSB cleaves upstream of this single nucleotide of homology between the repeats.

In the parental cell line, we found that the frequency of RMR events restoring GFP was highest for the 201 nt repeat, and decreased with the length of the repeat (Fig 4A). Indeed, such repair using the 51 nt repeat was largely undetectable. Therefore, inducing a DSB with non-homologous sequences on both sides of the repeats causes a greater requirement for a longer repeat to induce RMR events. We then analyzed the influence of RAD52 on RMR events using the mid-ins DSB, and found that RAD52 complementation promoted these events for each of the repeat lengths (i.e., 201, 141, 102, and 73 nt repeats, Fig 4B, S3D Fig). We found similar results for RAD52 complementation in the *RAD52*^*KO*^*POLQ*^*e16m*^ cell line, and RNAi depletion of RAD52 (siRAD52 treatment), whereas POLQ complementation did not promote these events (Fig 4B). Notably, overexpression of RAD52 in the parental cells also promoted RMR events induced by the mid-ins DSB with the 201, 141, 102 nt repeats, but not any of the other RMR events (S4A Fig). This finding indicates that the level of RAD52 is a limiting factor for RMR events with repeats flanked by non-homologous sequences. In summary, these results with the mid-ins DSB, combined with the above finding (Fig 3B) that RAD52 has a greater effect on 50 nt RMR events that are induced by the 5' DSB vs. excision of the non-homologous sequence (i.e. the 5' & 3' edge DSBs), indicate that RAD52 is particularly important for RMR events that involve removal of a non-homologous sequence.

### POLQ is important for oligonucleotide microhomology-templated DSB repair events

**Fig 5:**
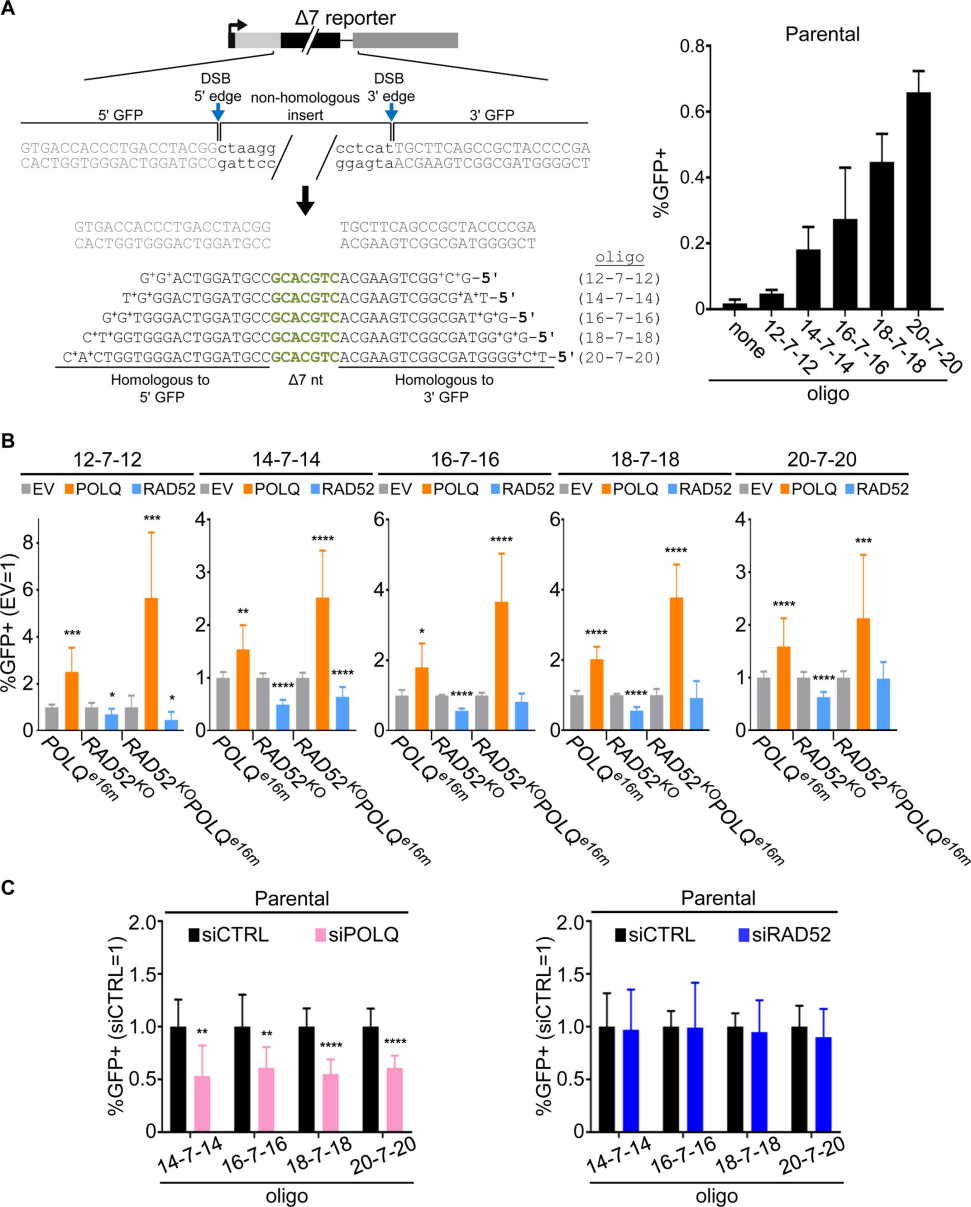
POLQ promotes oligonucleotide microhomology-templated repair events requiring nascent DNA synthesis. (A) Diagram of Δ7 reporter in which 7 nt have been deleted from the 3' GFP sequence. The GFP coding sequence can be restored via templated insertion of the omitted 7 nt, which is provided by transfection of oligonucleotides. Five different oligonucleotides were used, which contain these 7 nt flanked on both sides by 12, 14, 16, 18, or 20 nt of homology to the 5' and 3' GFP sequences (12-7-12, 14-7-14, 16-7-16, 18-7-18, or 20-7-20, respectively). The plus signs indicate phosphorothioate linkages. These events are induced by expression of two sgRNAs and Cas9 to target DSBs at the edges of the 5' and 3' GFP sequences, thereby excising the non-homologous insert. These transfections also included the oligonucleotide templates and empty vector (EV). Shown are the percentages of GFP+ cells from these transfections, normalized to transfection efficiency, either in the absence (none) or presence of the 12-7-12, 14-7-14, 16-7-16, 18-7-18, or 20-7-20 oligonucleotides. Two independent clones were tested for each reporter with four independent replicates for a total *n* = 8, and error bars represent SD. (B) Influence of RAD52 and POLQ on repair events using oligonucleotide templates. *RAD52*^*KO*^, *POLQ*^*e16m*^, and *RAD52*^*KO*^*POLQ*^*e16m*^ Δ7 reporter cell lines were transfected as in (A), but including EV, RAD52 expression vector, or POLQ expression vector. GFP+ frequencies are normalized to transfection efficiency and shown relative to EV (EV = 1). Error bars represent SD. Two independent clones were tested for each reporter. *n* = 8 for 12-7-12, 14-7-14, 16-7-16, and *n* = 16 for 18-7-18 and 20-7-20. † *P* < 0.05 (unadjusted P-value), * *P* < 0.05, ** *P* < 0.01, *** *P* < 0.005, **** *P* < 0.001, EV vs. complementation, using unpaired *t*-test with Holm-Sidak correction. (C) The parental Δ7 reporter cell line was examined with siRNA treatments as in Fig 3. Two independent clones were tested for each treatment with four independent replicates for a total *n* = 8, and error bars represent SD. ** *P* < 0.01, **** *P* < 0.001, siCTRL vs. siRNA treatment using unpaired *t*-test with Holm-Sidak correction.

Since POLQ appears important only for the RMR event using the 6 nt repeat, we considered that POLQ might also be important for other repair events. In particular, we considered that POLQ might be important for DSB repair events that require nascent DNA synthesis. We based this hypothesis on previous studies showing that POLQ mediates annealing of oligonucleotides using short complementary ssDNA to template nascent DNA synthesis [19, 24]. To examine events that require nascent DNA synthesis, we modified our chromosomal RMR reporter system by deleting 7 nt from the 5' edge of the 3' GFP segment (Δ7 reporter; Fig 5A). Repair using an oligonucleotide with microhomology as a template that contains the missing 7 nt would restore GFP expression (i.e., oligonucleotide microhomology-templated repair). We used oligonucleotides that contained the missing 7 nt, which are flanked by equal lengths of homology to both the 5' and 3' GFP sequences, using several different lengths: 12, 14, 16, 18, or 20 nt (referred to as 12-7-12, 14-7-14, 16-7-16, 18-7-18, and 20-7-20, respectively, Fig 5A). The oligonucleotides also contain phosphorothioate linkages at the two terminal bases at both ends to promote stability [36]. These oligonucleotides were co-transfected with the sgRNA/Cas9 plasmids to induce DSBs at the edge of the 5' GFP and 3' GFP segments (i.e., the 5' & 3' edge DSBs, as described above). Using the parental U2OS cells, we found that each of the oligonucleotides induced GFP+ cells, which increased in frequency with the length of the flanking sequence homology (Fig 5A). To confirm the restoration of GFP in the Δ7 reporter with each of the oligonucleotides, the cells were sorted to enrich for GFP+ cells, and examined by PCR and sequencing (S2B Fig). We also confirmed that both the 5' and 3' DSBs are required to induce these oligonucleotide microhomology-templated events (S4B Fig).

We then analyzed the role of RAD52 and POLQ on the Δ7 reporter assay using each of the oligonucleotide templates. We performed both complementation and RNAi analysis, although since the 12-7-12 oligonucleotide events were near background levels, we found it difficult to examine effects of RNAi in potentially reducing these events (S4C Fig). In any case, both types of analysis were feasible for the rest of the oligonucleotides (14-7-14 and longer). From both complementation and RNAi analysis, we found that RAD52 was dispensable for such repair with each of the oligonucleotides (Fig 5B and 5C and S4B and S4D Fig). In contrast, we found that POLQ expression in the *POLQ*^*e16m*^ cells significantly promoted the induction of GFP+ cells using all of the oligonucleotides (Fig 5B and S4B and S4D Fig). POLQ expression had a similar effect on *RAD52*^*KO*^*POLQ*^*e16m*^ cells, and the fold-effects were magnified (Fig 5B and S4D Fig). Importantly, and consistent with the complementation analysis, siPOLQ treatment caused a reduction in each of these events (Fig 5C, i.e., with the 14-7-14 oligonucleotide and longer).

To provide a contrast for these assays, we also examined end joining (EJ) events that do not require annealing of a homologous repeat or nascent DNA synthesis. Specifically, we used EJ7ins (S5A Fig), in which the non-homologous insert is flanked by the first two bases (GG) and the final base (C) of the GGC codon for Glycine 67 for GFP. Following DSBs to excise the non-homologous insert, EJ without indels between the distal DSBs would restore the GGC codon. Thus, restoration of GFP+ does not involve any nascent DNA synthesis nor annealing of microhomology. This assay is a variant of EJ7-GFP [30]; the only difference is the size of the non-homologous insert. We also performed experiments with an oligonucleotide that is homologous to the EJ junction that could possibly bridge the DSB ends during repair. Specifically, we used an oligonucleotide with 14 nt of homology to each side of the EJ junction, but with no bases in between (i.e., 14-0-14, S5A Fig). We included this experiment to provide a contrast to the Δ7 reporter assays, which use oligonucleotides to template nascent DNA synthesis. We found that including the 14-0-14 oligonucleotide did not promote the EJ event measured by EJ7ins, compared to a control oligonucleotide (luciferase/LUC), or to transfections without any oligonucleotide (S5A Fig).

**Fig 6:**
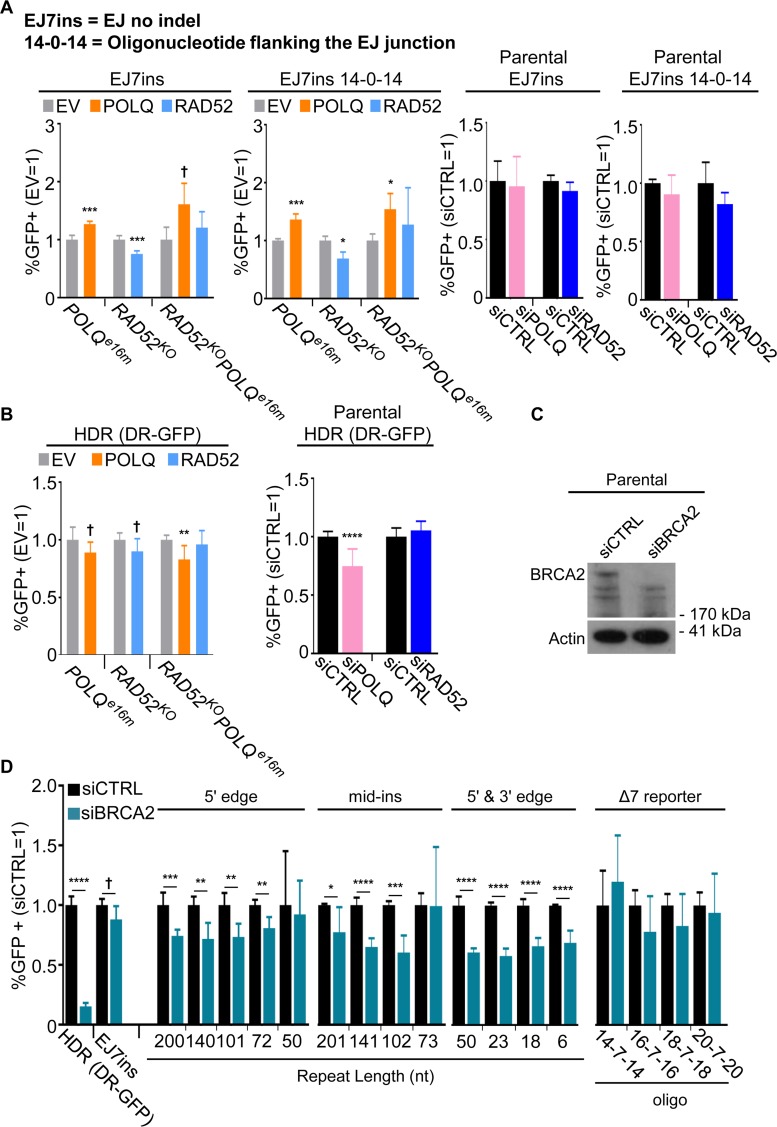
POLQ modestly affects EJ repair and HDR, whereas BRCA2 promotes HDR to a much greater degree than RMR events. (A) Influence of POLQ and RAD52 on EJ repair events induced by sgRNA/Cas9-mediated DSBs targeted to the 5' edge and 3' edge of the non-homologous insert (5' & 3' edge) in the EJ7ins reporter (S5 Fig). In this reporter, restoration of GFP expression occurs via EJ without indels. Shown are the percentages of GFP+ cells normalized to both transfection efficiency and to parallel EV samples (EV = 1). The *RAD52*^*KO*^, *POLQ*^*e16m*^, and *RAD52*^*KO*^*POLQ*^*e16m*^ EJ7ins reporter cells were transfected with expression vectors for the sgRNAs and Cas9, along with empty vector (EV), POLQ expression vector, or RAD52 expression vector, and parallel reactions that contained an oligonucleotide that had 14 nt of homology to the 5' and 3' GFP sequences (14-0-14) were tested for possible roles of oligonucleotide bridging of DSB ends. Two independent clones were tested for each cell line with two independent replicates for a total *n* = 4, and error bars represent SD. Experiments with siRNA were performed as in Fig 3. Two independent clones were tested for each reporter with two independent replicates for a total *n* = 4. Error bars represent SD. † *P* < 0.05 (unadjusted P-value), * *P* < 0.05, ** *P* < 0.01, *** *P* < 0.005, **** *P* < 0.001, EV vs. complementation, using unpaired *t*-test with Holm-Sidak correction. (B) Influence of RAD52 and POLQ on the HDR reporter (DR-GFP). *RAD52*^*KO*^, *POLQ*^*e16m*^, and *RAD52*^*KO*^*POLQ*^*e16m*^ cells with stable transfection of DR-GFP were transfected with an sgRNA and Cas9 expression plasmid targeting DR-GFP, along with EV, RAD52 expression vector, or POLQ expression vector. Experiments with siRNA were performed as in (A). Error bars represent SD and *n* = 8. † *P* < 0.05 (unadjusted P-value), ** *P* < 0.01, **** *P* < 0.001, EV vs. complementation, and siCTRL vs. siRNA treatment using unpaired *t*-test with Holm-Sidak correction. (C) Immunoblot analysis shows the depletion of BRCA2 with siRNA in the parental line using siCTRL or a pool of four BRCA2 siRNAs (siBRCA2). Actin was a loading control. (D) Influence of BRCA2 depletion on chromosomal break repair events. Parental cells with each reporter were co-transfected with the sgRNA and Cas9 expression plasmid as indicated, along with siCTRL or siBRCA2. For the Δ7 reporter, oligonucleotides were co-transfected as indicated. GFP+ frequencies are normalized to transfection efficiency and shown relative to the non-targeting siRNA (siCTRL = 1). *n* = 4 for 5' & 3' edge, *n* = 6 for 5' edge and mid-ins, and *n* = 8 for Δ7 reporter. Error bars represent SD. † *P* < 0.05 (unadjusted P-value), * *P* < 0.05, ** *P* < 0.01, *** *P* < 0.005, **** *P* < 0.001, siCTRL vs. siBRCA2, using unpaired *t*-test with Holm-Sidak correction.

We then examined the influence of POLQ and RAD52 on these EJ events. We found that siPOLQ and siRAD52 treatments did not cause a decrease in the frequency of such EJ events, with or without the 14-0-14 bridging oligonucleotide (Fig 6A). From analysis of the mutant cell lines, expression of POLQ from the complementation vector caused a modest increase in these EJ events, irrespective of whether an oligonucleotide was included (Fig 6A, S5B Fig). Notably, these effects of the POLQ complementation vector on EJ were less than for the oligonucleotide microhomology-templated repair events (Figs 5B and 6A, in the *POLQ*^*e16m*^ cells, EJ7ins promoted ≤1.36-fold, whereas the Δ7 reporter promoted between 1.6 to 2.6-fold, depending on the oligonucleotide). Furthermore, as mentioned above, the oligonucleotide microhomology-templated events (Fig 5C), but not the EJ events (Fig 6A), were reduced by siPOLQ treatment. Altogether these findings indicate that POLQ promotes oligonucleotide microhomology-templated repair to a greater degree than EJ without use of microhomology.

For another contrast to the above DSB repair events, we also examined HDR, using the DR-GFP reporter, which measures use of a homologous sequence as a template for gene conversion [37]. For these experiments, we used Cas9 and an sgRNA to induce the DSB in DR-GFP [38]. We found that neither RAD52 nor POLQ complementation vectors caused an increase in HDR in the respective mutant cell lines (Fig 6B, S5D Fig). Similarly, siRAD52 treatment did not cause a decrease in HDR, although siPOLQ caused a modest decrease in HDR (Fig 6B, 1.3-fold). These findings indicate that RAD52 and POLQ do not have a substantial role in HDR, as measured using the DR-GFP reporter.

### Influence of BRCA2 on RMR and oligonucleotide microhomology-templated repair events

To provide a contrast with RAD52 and POLQ, we also examined the influence of BRCA2 on several DSB repair events. BRCA2 is important for RAD51 recruitment to DNA damage and HDR [39]. We first sought to confirm that BRCA2 is important for HDR using the DR-GFP reporter [37], using siRNAs targeting BRCA2 (siBRCA2, depletion of BRCA2 validated by immunoblotting, Fig 6C). As expected, we found that siBRCA2 treatment caused a marked decrease in HDR (Fig 6D). We then examined the RMR reporter assays, and found that siBRCA2 treatment caused a decrease in nearly all of the RMR events (Fig 6D). Accordingly, BRCA2 appears to promote RMR events irrespective of the repeat length or DSB induced (Fig 6D), which is distinct from the results with RAD52 and POLQ. Finally, siBRCA2 treatment did not have a substantial effect on EJ (EJ7-ins reporter), nor the oligonucleotide microhomology-templated events (the Δ7 reporter, Fig 6D). In summary, BRCA2 promotes several RMR events, but to a much lesser degree than its requirement for HDR.

### Combined disruption of RAD52 and POLQ causes a synthetic reduction in replication fork restart velocity

**Fig 7:**
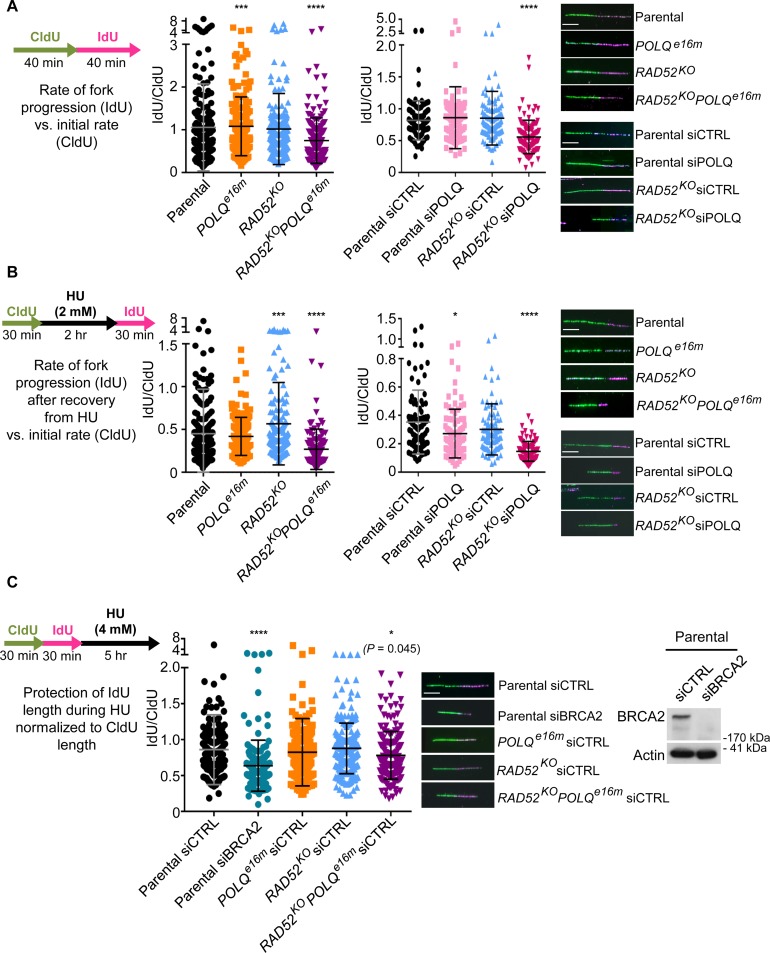
Combined disruption of RAD52 and POLQ causes a synthetic reduction in replication fork restart velocity. (A) Replication fork progression without stress. Parental, *POLQ*^*e16m*^, *RAD52*^*KO*^, and *RAD52*^*KO*^*POLQ*^*e16m*^ cells were pulse labeled with CldU followed by IdU prior to fiber analysis. Additionally, parental and *RAD52*^*KO*^ cells were treated with siPOLQ or siCTRL (same siRNAs as in Fig 1D) and were pulsed labeled with CldU followed by IdU prior to fiber analysis. Shown are representative DNA fibers and quantification of the DNA fiber lengths represented as an IdU/CldU ratio. More than 240 fibers were analyzed for each condition. * *P* < 0.05, *** *P* < 0.005, **** *P* < 0.001, parental vs. mutant, and parental siCTRL vs. other siRNA treatment using the Kolmogorov-Smirnov test to analyze DNA fiber lengths. Bar = 10 μm. (B) Replication fork restart after replication stress. Cell lines in (A) were pulse labeled with CldU, treated with HU, and pulse labeled with IdU prior to fiber analysis, shown as in (A). Numbers of fibers analyzed, replicates, and statistics are as in (A). (C) Protecting replication forks from degradation during replication stress. Parental cells were treated with a pool of four BRCA2 siRNAs (siBRCA2) or siCTRL. Immunoblot analysis shows the depletion of BRCA2, with Actin as a loading control. *RAD52*^*KO*^, *POLQ*^*e16m*^, and *RAD52*^*KO*^*POLQ*^*e16m*^ cells were treated with siCTRL to enable direct comparisons. Following siRNA treatment, cells were pulse labeled with CldU, followed by IdU, and then exposed to HU for 5 hr, prior to fiber analysis, shown as in (A). Numbers of fibers analyzed and statistics are as in (A).

Disruption of *BRCA1* and *BRCA2* causes defects not only in HDR, but also the cellular response to replication stress [6]. Thus, we next examined whether disruption of *RAD52* and *POLQ* may also affect replication stress responses, using DNA fiber analysis [40]. We first examined how the disruption of *RAD52* and/or *POLQ* would affect the rate of replication fork progression in unstressed cells. Specifically, we pulse labeled cells with the thymidine analog CldU, followed by a pulse label with the thymidine analog IdU for equal amounts of time (40 min). Antibodies against each analog that are conjugated to different fluorophores allowed for the visualization of the fibers. We measured the lengths of the labels for individual fibers to calculate the IdU/CldU ratio, and thereby measure the rate of fork progression, which we refer to as replication fork velocity (Fig 7A). We found that the *POLQ*^*e16m*^ cells showed a modest but significant increase in replication fork velocity, whereas disruption of *RAD52* had no effect (Fig 7A). In contrast, the *RAD52*^*KO*^*POLQ*^*e16m*^ cells showed a significant reduction in replication fork velocity (Fig 7A). Similarly, siPOLQ treatment caused a reduction in replication fork velocity in the *RAD52*^*KO*^ cells, but not parental cells (Fig 7A). As described above, depletion of POLQ mRNA via siPOLQ was confirmed in both parental and *RAD52*^*KO*^ cells (Fig 1D). We also examined the fraction of stalled replication forks (i.e., CldU-labeled fibers only). We found that *POLQ*^*e16m*^ and *RAD52*^*KO*^*POLQ*^*e16m*^ cells, but not *RAD52*^*KO*^ cells nor siPOLQ treated cells, showed a modest decrease in the frequency of stalled replication forks (S6A Fig).

We next examined the influence of RAD52 and POLQ on the restart of replication forks after replication stress. In this analysis, cells were pulse labeled with CldU, and then treated with hydroxyurea (HU), which causes a depletion of dNTPs, thereby causing replication fork stalling [40]. Following release from HU, cells were pulse labeled with IdU, and the DNA fibers were analyzed for the IdU/CldU ratio to measure the rate of replication restart, which we refer to as replication restart velocity (Fig 7B). We also quantified the frequency of stalled replication forks (S6A Fig). We found that replication fork restart velocity was not distinct between the *POLQ*^*e16m*^ cell line and the parental cells line, but was higher in the *RAD52*^*KO*^ vs. parental (Fig 7B). Strikingly, the *RAD52*^*KO*^*POLQ*^*e16m*^ cell line showed a marked decrease in replication fork restart velocity, compared to the parental cell line (Fig 7B). Similarly, siPOLQ treatment in the *RAD52*^*KO*^ cell line caused a marked decrease in replication fork restart velocity, whereas siPOLQ treatment only caused a modest decrease in the parental cell line (Fig 7B). Apart from fork velocity, we did not observe major effects on the percentage of stalled replication forks, apart from a modest increase with the *RAD52*^*KO*^*POLQ*^*e16m*^ cell line (S6B Fig).

We also examined replication fork protection during stalling, which has been shown to require BRCA2, among other factors [5, 41]. In this analysis, cells are pulse labeled with CldU, followed by IdU, and then treated with HU for 5 hr [5, 41]. To begin with, we examined cells treated with siRNAs targeting BRCA2, and consistent with prior studies, found that depletion of BRCA2 (confirmed by immunoblotting) causes a reduction in the IdU/CldU ratio, reflecting fork degradation [5, 41] (Fig 7C). In contrast, BRCA2 depletion did not cause an obvious effect on the IdU/CldU ratio when the HU treatment was positioned between the two labels (S6B Fig). Regarding the influence of RAD52 and POLQ on fork protection during stalling, we found that the *POLQ*^*e16m*^ and *RAD52*^*KO*^ cell lines were not distinct from the parental cell line (Fig 7C). The *RAD52*^*KO*^*POLQ*^*e16m*^ cells showed a modest decrease in the IdU/CldU ratio in these experiments (*P* = 0.045, Fig 7C), similar to the findings without replication stress (see Fig 7A). Taken together, these finding indicate that the combined disruption of *RAD52* and *POLQ* causes a significant decrease in the velocity of replication fork progression, particularly during restart of stalled replication forks, but does not have an obvious effect on protection of stalled replication forks from degradation.

## Discussion

As RAD52 and POLQ are each synthetic lethal targets for cells deficient in BRCA1 and BRCA2 [8–10], we have sought to test whether RAD52 and POLQ have distinct vs. redundant functions in chromosomal break repair, sensitivity to genotoxins, and/or response to replication stress. Beginning with genotoxin sensitivity, we found that disruption of *RAD52* and *POLQ* each caused hypersensitivity to cisplatin, and combined disruption of these factors caused an at least additive hypersensitization. Accordingly, RAD52 and POLQ appear to have non-epistatic roles in cisplatin resistance. We also found that RAD52 and POLQ have different effects on DSB repair, using a series of novel assays for RMR and oligonucleotide microhomology-templated repair events. The DSB reporter analysis involved multiple approaches to examine RAD52 and POLQ, i.e, both complementation analysis in mutant cell lines, and RNAi. We suggest that identifying DSB repair phenotypes that are relatively consistent between these approaches, and that reveal patterns among multiple reporter contexts, has provided insight into the influence of RAD52 and POLQ on such DSB repair events.

Beginning with RAD52, we found that this factor is important for RMR events using ≥ 50 nt, and when repeat sequences also require removal of non-homologous sequence flanking at least one of the repeats. The influence of RAD52 on events with this range of repeat length is consistent with biochemical properties of RAD52. In particular, single-molecule studies have shown that multimeric rings of RAD52 interact with ssDNA by optimally binding ~30 nt around the protein ring [12, 16, 42–44]. Regarding removal of non-homologous sequences flanking a region of homology, other studies also support a role of RAD52 in such events. For example, our laboratory recently reported that an RMR event in mouse cells requiring removal of several kb of non-homologous sequence was particularly dependent on RAD52 [45], and another recent study showed that HDR events requiring removal of a non-homologous sequences were also promoted by RAD52 [46]. Thus, we suggest that RAD52 may have a specific role in synapsis of ≥ 50 nt of homology that is embedded within a non-homologous sequence, and thereby stabilize this intermediate to facilitate cleavage of the non-homologous sequence to complete repair.

For POLQ, we found that this factor was important for RMR events using 6 nt, but not ≥ 18 nt, as well as DSB repair events requiring nascent DNA synthesis from oligonucleotide templates with 12–20 nt of microhomology. These findings are consistent with studies of POLQ-dependent extension of oligonucleotide substrates that are annealed via a very short (e.g., 4 nt) sequence [17, 19]. This activity of POLQ is consistent with the structure of its C-terminal polymerase domain, which contains additional insertions loops that are not found in other A-family DNA polymerases [18]. Within these unique insertions loops, multiple residues facilitate specific interactions with the primer strand, which appear to enable extension of minimally annealed DNA substrates [17, 18, 47]. Notably, combined loss of POLQ and RAD52 did not reveal any synthetic defects in DSB repair events (e.g., repair events promoted by POLQ were the same in the *POLQ*^*e16m*^ cells as the *POLQ*^*e16m*^*RAD52*^*KO*^ cells), which altogether indicate that these factors have distinct roles in such repair.

We also found that RMR events involving 18–23 nt of homology were unaffected by RAD52 and POLQ. Notably, events with ≤ 23 nt of homology are nearly undetectable if the repeat is flanked by a non-homologous sequence. Accordingly, the mechanisms that mediate such RMR events with ≤ 23 nt of homology may be insufficient to facilitate cleavage of a non-homologous tail. Alternatively, ≤ 23 nt of homology may not be sufficient to compete with shorter lengths of homology that are closer to the DSB end. In any case, other factors besides RAD52 and POLQ appear to be sufficient to mediate RMR events involving 18–23 nt of homology. Indeed, beyond these particular repair events, we suggest that other factors are likely involved in RMR events of diverse repeat lengths, since each RMR event we examined remains readily detectable in cells deficient in RAD52 and/or POLQ.

The factors apart from RAD52 and POLQ that mediate RMR events remain unclear. Although, we found that BRCA2 mediates several RMR events at multiple repeat lengths (i.e., 201 nt– 6 nt), which is distinct from our findings with RAD52 and POLQ. However, the influence of BRCA2 on these RMR events was markedly lower than its influence on HDR. Furthermore, in other studies, BRCA2 has been shown to suppress RMR events, likely due to competition with HDR [26, 48]. However, BRCA2-mediated HDR may not be a substantial competitive pathway for the RMR events measured here. Namely, the DSBs in these assays are not readily repaired by HDR, which requires a repair template with homology on both sides of the DSB. Nevertheless, our findings support the notion that BRCA2 has a distinct role in DSB repair vs. POLQ and RAD52, since BRCA2 is required for HDR, whereas POLQ and RAD52 do not appear to have substantial roles in HDR.

Consistent with BRCA2 having distinct roles in genome maintenance vs. POLQ and RAD52, these factors differentially affect the response to replication stress. As in other studies [5, 41], we found that depletion of BRCA2 caused a defect in protecting stalled replication forks from degradation, but did not cause obvious effects on the restart of stalled forks. In contrast, disruption of *POLQ* and *RAD52*, either alone or in combination, caused no major effects on protection of stalled replication forks, using the same experimental conditions that reveal a role for BRCA2. We also found that disruption of *RAD52* or *POLQ* individually did not obviously cause defects in the frequency of restart of stalled replication forks. These findings are consistent with other studies of RAD52, in which this factor appears dispensable for restart of stalled replication forks, but rather appears important for restart of collapsed forks (i.e., following long-term HU treatment) [49]. Although, a recent report found that combined treatment of a small molecule that targets RAD52, along with a CDC7 inhibitor, caused an increase in the frequency of stalled replication forks after HU treatment [50]. Furthermore, our findings with POLQ are distinct from a report that cells depleted of POLQ via RNAi show an increase in the frequency of collapsed forks following recovery from HU [9].

Nevertheless, we found that combined disruption of POLQ and RAD52 caused a marked decrease in replication fork restart velocity, as indicated by a substantial reduction in the length of the labeled DNA fiber after release from HU. The cause of this effect on fork restart velocity could be due to several mechanisms. For example, RAD52 could promote an annealing intermediate important for stabilizing the stalled fork, and/or re-establishing the replisome [49]. Indeed, a recent report found that RAD52 is important to suppress excessive ssDNA formation at stalled forks [50]. Similarly, POLQ could stabilize the stalled fork via its primer extension activity [51]. RAD52 or POLQ could also recruit other factors important for these processes [47, 52]. Alternatively, loss of one of these factors could cause accumulation of an intermediate that requires the other factor for resolution to enable rapid fork restart. Along these lines, disruptions of POLQ and/or RAD52 may affect other aspects of DNA replication that may not have been revealed in our analysis, such as suppressing fork discontinuities [50, 53], which could contribute to the reduced fork velocity that we observed. In summary, these findings indicate that RAD52 and POLQ have distinct roles in genome maintenance, including DSB repair and replication fork restart velocity. Since these factors are emerging therapeutic targets [8–11], these findings indicate that combined disruption of these factors may be an effective approach for genotoxin sensitization and/or synthetic lethality strategies.

## Materials and methods

### Cell lines and reporter plasmids

All sgRNAs, primers, and oligonucleotide template sequences are found in S1 Table. The parental cell line in this study is the human osteosarcoma U2OS Flp-In T-REx cell line, which is stably transfected with pFRT/lacZeo [29, 30]. Cells were cultured as previously described [54], and using the Lonza MycoAlert PLUS Mycoplasma Detection Kit, cell lines tested negative for mycoplasma contamination. To generate plasmids for inducing DSBs, sgRNA sequences were cloned into the px330 vector (Addgene #42230) that expresses an sgRNA and Cas9 [55]. To generate the mutant cell lines, these sgRNA/Cas9 plasmids were co-transfected (400 ng of each sgRNA vector) with the dsRED expression plasmid (120 ng) and 3.6 μl of Lipofectamine 2000. After 3 days, the cells were sorted (using an Aria 3 or Aria SORP, Becton Dickinson) to enrich for dsRED-positive transfected cells followed by low-density plating. To generate the *POLQ*^*e16m*^ cell line, two sgRNAs were used to target exon 16 of *POLQ*, and clones were screened by PCR amplification and sequencing. For the *RAD52*^*KO*^ cell line, two sgRNAs were used to target exon 3 and exon 9 of *RAD52* [49]. The *RAD52*^*KO*^*POLQ*^*e16m*^ cell line, was generated in the *POLQ*^*e16m*^ cell line using the *RAD52* exon 3 sgRNA and an sgRNA that targets *RAD52* exon 4. The *RAD52*^*KO*^ and *RAD52*^*KO*^*POLQ*^*e16m*^ cell lines were identified by screening individual clones using RAD52 immunoblot analysis.

The RMR200 reporter plasmid was generated by inserting two gBLOCK fragments (IDT) into the pcDNA5-FRT-EJ7-GFP vector [30]: 1) a non-homologous sequence derived from the puromycin-resistance gene [54] to generate the EJ7-ins reporter, and 2) the 3' GFP fragment, which contains 200 nt of homology to the 5' GFP sequence. This RMR200 reporter plasmid was used to generate the variants with the different 3' repeat sequences. These RMR reporter plasmids (100 ng) were integrated into the U2OS Flp-In T-REx cells by co-transfection with the PGK-Flp vector (400 ng) [35], using Lipofectamine 2000 (Thermofisher) as described below for the DSB reporter assays. Integrated clones were selected using hygromycin (0.2 μg/μl), and subsequently screened with PCR analysis to confirm integration (S1B, S1C Fig). To integrate the DR-GFP reporter into the parental U2OS Flp-In T-Rex cell lines, and the various mutant cell lines, 10 μg of XhoI linearized Pim-DRGFP plasmid [54] was electroporated into each cell line (0.8 ml volume), followed by selection of stably transfected cells in 0.8 μg/ml puromycin, which were pooled together for analysis. The RNAi experiments to examine HDR used the previously described U2OS DR-GFP reporter cell line [54].

### Cell cycle analysis

Cell cycle analysis was performed as previously described [45]. Briefly, the cells were pulse labeled with BrdU (BD Pharmingen, 51-2420KC) for 30 min at 37°C. The cells were then fixed with 70% ethanol, and stained with FITC-conjugated anti-BrdU (BD Pharmingen, 51-33284X), followed by and propidium iodide (Sigma, P4170) supplemented with RNase (Sigma, R4642) for 30 min at 37°C. Each sample was analyzed by flow cytometry using a CyAn-ADP (Dako).

### DSB reporter assays

Cells with integrated reporter cassettes were seeded at 0.5 x 10^5^ cells per well (24 well plate). The following day, the cells were transfected with 200 ng of each sgRNA/Cas9 vector and 1.8 μl of Lipofectamine 2000 with 0.5 ml of antibiotic-free media. To normalize the frequency of repair events between experiments, parallel transfections with GFP expression vector (200 ng, pCAGGS-NZE-GFP [54]) were included. In the RAD52 complementation experiments, the reactions were performed as describe above with the addition of 25 ng of empty vector (pCMV6-XL5) or RAD52 expression vector (Origene RC238113). For the POLQ complementation, 100 ng of empty vector (pCAGGS-BSKX) [56] or POLQ expression vector [57] was added to the reactions. Similar transfections with equivalent concentrations of expression vectors were used to generate samples for immunoblotting analysis. For RAD52 and POLQ complementation in the double mutant cell line, additional empty vector (pCAGGS-BSKX) was included to ensure an equivalent amount of total plasmid in each transfection. For the Δ7 reporter, transfections were scaled 2-fold onto a 12 well dish, and transfections were performed as described above with the addition of 10 nM (final concentration) of the indicated oligonucleotide to the reaction. Each oligonucleotide contained phosphorothioate linkages on the first two and last two terminal bases (IDT). In the experiments with siRNA, 5 pmol of either non-targeting siRNA (siCTRL; Dharmacon, D-001810-01-20) or a pool of four siRNAs targeting RAD52, POLQ, or BRCA2 (Dharmacon siGENOME siRNAs, sequences from manufacturer in S1 Table) was included in the respective Lipofectamine 2000 transfections. In addition, for POLQ siRNA experiments, the day before the above transfections with Lipofectamine 2000, cells were first treated with 5 pmol of either siCTRL or the four siRNAs targeting POLQ, using Lipofectamine RNAiMAX (Thermofisher). For immunoblotting analysis to confirm BRCA2 and RAD52 depletion, an equivalent concentration of cells and siRNA as for the reporter assays was used for a transfection with Lipofectamine RNAiMAX. For each reporter assay, three days after transfection, the percentage of GFP+ cells were determined by flow cytometry using a CyAn-ADP (Dako), as previously described [54]. The repair value for each sgRNA(s)/CAS9 transfection was first normalized to transfection efficiency using the parallel transfection with a GFP expression vector. For comparisons vs. EV or siCTRL, each repair value normalized to transfection efficiency was divided by the mean repair value for the parallel control transfections (i.e., siCTRL and/or EV). To confirm the sequence of GFP+ products for each reporter, transfected parental cells were sorted (Aria III or Aria SORP, Becton Dickinson) to enrich for cells expressing GFP, which were analyzed by PCR-amplification and sequencing (S2 Fig).

### Clonogenic survival

Clonogenic survival was assessed by plating 10^3^ cells on 6 well plates in media containing cisplatin (1.0 or 3.0 μM, Pfizer) or Olaparib (0.75 or 1.5 μM, Selleckchem), or were untreated (equivalent volume of DMSO added as a control). For ionizing radiation, each cell line was exposed to 1.5 or 3 Gy (Gammacell 3000), or left untreated, prior to plating. Cells were cultured for 9 days, and stained with crystal violet (Sigma). Colonies of approximately 50 or more cells were quantified under a 10x objective, and fraction survival was calculated relative to the number of colonies on the untreated control wells that were plated in parallel. For experiments with siRNA depletion, 10^5^ cells were plated on a 12 well plate with either control siRNA (siCTRL) or a pool of four POLQ siRNAs (40 pmol; Dharmacon, sequences in S1 Table), using Lipofectamine RNAiMAX. Two days after transfection, cells were treated with genotoxins to test clonogenic survival, as described above.

### qRT-PCR analysis

To test for depletion of POLQ mRNA, cells were transfected on a 6 well dish with 20 pmol of siCTRL or pool of four POLQ siRNAs (see above) using Lipofectamine RNAiMAX (2 ml total volume). On the following day, cells were transfected with the respective siRNA (20 pmol) and two plasmids (400 ng of pgk-PURO and 1200 ng EV) [54], using Lipofectamine 2000 (2 ml total volume), as for the reporter assays. The day after transfection, cells were treated with puromycin (2 μg/ml concentration) for one day to enrich for transfected cells, and then RNA was isolated using the RNeasy Plus Minikit (Qiagen 74134). The RNA was treated with M-MLV Reverse Transcriptase (Promega M170A) to generate cDNA, which was amplified in an Applied Biosystems 7900HT Fast Real Time PCR system using SYBR-green, with the primer sequences shown in S1 Table.

### Immunoblot analysis

Immunoblotting analysis was performed by lysing the cells using NETN buffer (20 mM Tris pH 8, 100 mM NaCl, 1 mM EDTA, 0.5% IGEPAL, 1.25 mM DTT, and protease inhibitors, Roche) followed by several freeze-thaw cycles. The blots were probed with antibodies against: RAD52 1:500 (Santa Cruz Biotechnology, sc365341), FLAG 1:1000 (Sigma, A8592), BRCA2 1:1000 (Millipore, OP95-10006), or ACTIN 1:3000 (Sigma, A2066); and with the HRP-conjugated secondary antibodies rabbit anti-mouse 1:3000 (Abcam, ab205719) or goat anti-rabbit 1:3000 (Abcam, ab205718). ECL Western Blotting Substrate (Thermo Fisher Scientific, 32106) was used to detect HRP signal on film.

### DNA fiber analysis

For DNA fiber analysis, cells were plated at 10^5^ cells/well on a 6 well plate. The following day, the cells were pulse labeled with CldU (50 μM, Sigma C6891) for 40 min followed by IdU (250 μM, Sigma I7125) for 40 min. When testing replication stress recovery, the cells were pulse labeled with CldU for 30 min, hydroxyurea (2 mM) for 2 hr, then IdU for 30 min. In the fork protection assay the cells were pulse labeled with CldU for 30 min, IdU for 30 min, then hydroxyurea (4 mM) for 5 hr. In the experiments with siRNA, 10^5^ cells were plated on a 12 well plate with either control siRNA (siCTRL), a pool of four BRCA2 or POLQ siRNAs (40 pmol; Dharmacon, sequences in S1 Table), using Lipofectamine RNAiMAX, and the following day the cells were seeded on a 6 well plate. The next day (two days after transfection) cells were treated with the nucleotides and HU as above. DNA was isolated from cells using the FiberPrep DNA extraction kit (Genomic Vision, EXT-001). These DNA preparations were combed onto vinylsilane coated coverslips (Genomic Vision, COV-002-RUO) using the FiberComb Molecular Combing System (Genomic Vision, MCS-001). After combing, the coverslips were dehydrated, and then denatured using 0.5 M NaOH and 1 M NaCl. The coverslips were blocked with 5% BSA in PBS, followed by treatment with a rat antibody to detect the CldU signal and a mouse antibody to detect the IdU signal (1:50; Abcam ab6326 and BD Biosciences 347580, respectively), and then with goat anti-rat Alexa Fluor 488 and goat anti-mouse Alexa Fluor 555 (colored green and violet, respectively, by the image capture software to clearly distinguish the signals) (1:50; Thermo Fisher Scientific, A110060 and A28180, respectively). The coverslips were mounted using ProLong Gold Antifade (Thermo Fisher Scientific), and the slides were imaged using a Zeiss Observer II with a 40x oil immersion objective, and fiber lengths were quantified using Image J [58].

## Supporting information

S1 Fig GFP reporters, integration schematic, and confirmation PCR. (A). Diagram of GFP reporter cassettes. (B). Schematic of the FRT/Flp system used to introduce the reporter cassettes into a specific chromosomal FRT locus in U2OS cells. (C) PCR products from parental (U2OS) cells with integrated reporters using primers that flank the downstream FRT site (pcDNA5-Fwd with lacZ-Rev). Parental cells without any integrated reporters were used as a negative control, and primers that amplify Actin were used as a positive control.
(TIF)

S2 Fig PCR amplification of cells sorted to enrich for GFP+ cells. (A) PCR amplification products using primers that flank the GFP cassette (RMR1 and RMR2) for each reporter cell line after expressing the indicated sgRNA/Cas9 (5' edge and 5' & 3' edges), followed by cell sorting to enrich for GFP+ cells. (B) PCR amplification products using primers that flank the GFP cassette (RMR1 and RMR2) from the Δ7 reporter cassette with the indicated oligonucleotide and the EJ7ins reporter cassette, expressing the sgRNAs/Cas9 targeting the 5' & 3' edges of the non-homologous insert. UN, untransfected; +, GFP+ cells enriched by sorting. Primers that amplify Actin were used as a positive control.
(TIF)

S3 Fig Frequencies of RMR events from Fig 3 and Fig 4 complementation analysis, but also including the parental cell line analysis from Fig 2 and Fig 4, and results from mutant cell lines transfected without EV, each normalized to transfection efficiency. (A) Shown are frequencies for the parental and the *POLQ*^*e16m*^ cell line for RMR events induced with the 5’ edge DSB, and combination of 5’ and 3’ edge DSBs. Two independent clones were tested for each reporter in each cell line with four independent replicates for a total *n* = 8, except the parental 23 nt repeat where six independent clones were tested for *n* = 24. Error bars represent SD. * *P* < 0.05, ** *P* < 0.01, *** *P* < 0.005, **** *P* < 0.001, parental no EV vs. mutant (No EV), and mutant EV vs. complementation using unpaired *t*-test with Holm-Sidak correction. † *P* < 0.05 using unpaired *t*-test, but not significant when corrected for multiple comparisons (i.e., unadjusted *P*-value). (B) Shown are frequencies for the parental and the *RAD52*^*KO*^ cell line for RMR events induced with the 5’ edge DSB, and combination of 5’ and 3’ edge DSBs. Experiments were performed as in panel (A), except for the *RAD52*^*KO*^ 18 nt repeat where four independent clones were tested for *n* = 16. Statistics are as in (A). (C) Shown are frequencies for the parental and the *RAD52*^*KO*^*POLQ*^*e16m*^ cell line for RMR events induced with the 5’ edge DSB, and combination of 5’ and 3’ edge DSBs. Experiments and statistics were performed as in (A). (D) Shown are frequencies for the parental, *RAD52*^*KO*^, and *RAD52*^*KO*^*POLQ*^*e16m*^ cell lines for RMR events induced with the mid-ins DSB. Experiments and statistics were performed as in (A).
(TIF)

S4 Fig (A) Frequencies of RMR events from overexpression of POLQ and RAD52 in the parental cell line, normalized to transfection efficiency. The parental reporter cell lines were transfected with an expression vector for the sgRNA(s) and Cas9, as indicated, along with empty vector (EV), POLQ expression vector, or RAD52 expression vector. Error bars represent SD. Two independent clones were tested for each reporter with two independent replicates for a total *n* = 4. † *P* < 0.05 (unadjusted *P*-value), * *P* < 0.05, ** *P* < 0.01, EV vs. overexpression (POLQ or RAD52) using unpaired *t*-test with Holm-Sidak correction. (B) Overexpression of POLQ and RAD52 in the parental Δ7 reporter cell line. The cell lines were transfected with an expression vector for the sgRNA(s) and Cas9, and empty vector (EV), POLQ expression vector, or RAD52 expression vector, along with the 12-7-12, 14-7-14, 16-7-16, 18-7-18, or 20-7-20 oligonucleotides. Frequencies of GFP+ cells analyzed as in (A). Error bars represent SD. Two independent clones were tested with two replicates for a total *n* = 4, except parental EV where four replicates were analyzed for *n* = 8. † *P* < 0.05 (unadjusted *P*-value), * *P* < 0.05, **** *P* < 0.001, EV vs. overexpression using unpaired *t*-test with Holm-Sidak correction. Also shown are the percentages of GFP+ cells when targeting sgRNA(s) and Cas9 to the 5' edge, 3' edge, or 5' & 3' edge in the parental Δ7 reporter cell line with the 12-7-12, 14-7-14, 16-7-16, 18-7-18, or 20-7-20 oligonucleotides. Error bars represent SD. Two independent clones were tested with two independent replicates for a total *n* = 4, except DSB 5' & 3' edge where four independent replicates were analyzed for *n* = 8. (C) Percentages of GFP+ cells from the non-targeting siRNA (siCTRL) in Fig 5C (left panel) normalized to transfection efficiency including the 12-7-12, 14-7-14, 16-7-16, 18-7-18, and 20-7-20 oligonucleotides. UN, untransfected. Error bars represent SD. Two independent clones were tested with two replicates for a total *n* = 4. (D) Percentages of GFP+ cells from Fig 5B complementation analysis, normalized to transfection efficiency, but also including the parental cell line with EV. Error bars represent SD. *n* = 8 for 12-7-12, 14-7-14, 16-7-16, and *n* = 16 for 18-7-18 and 20-7-20. † *P* < 0.05 (unadjusted P-value), * *P* < 0.05, ** *P* < 0.01, *** *P* < 0.005, **** *P* < 0.001, EV vs. complementation using unpaired *t*-test with Holm-Sidak correction.
(TIF)

S5 Fig (A) Diagram of EJ7ins reporter in which targeted DSBs by sgRNAs and Cas9 to excise the non-homologous insert can restore GFP expression via EJ without indels. Shown are the percentages of GFP+ cells in the parental EJ7ins reporter cell line. Cells were transfected with expression vectors for the sgRNAs and Cas9, along with empty vector (EV), either in the absence (none) or presence of an oligonucleotide that contained 14 nt of homology to the 5' and 3' GFP sequences (14-0-14) or a non-homologous control oligonucleotide (LUC, 28 nt total sequence). The plus signs indicate phosphorothioate linkages. Error bars represent SD. Two independent clones were tested with two independent replicates for a total *n* = 4. ns, not significant, 14-0-14 vs. no oligonucleotide (none) and LUC-oligo using unpaired *t*-test with Holm-Sidak correction. (B) Percentages of GFP+ cells from Fig 6A complementation analysis, normalized to transfection efficiency but including parental EV. Number of cell lines tested and number of replicates as in (A). Error bars represent SD. † *P* < 0.05 (unadjusted P-value), * *P* < 0.05, ** *P* < 0.01, **** *P* < 0.001, parental EV vs. mutant EV, and mutant EV vs. complementation using unpaired *t*-test with Holm-Sidak correction. (C)Percentages of GFP+ cells from Fig 6B complementation analysis, normalized to transfection efficiency but including parental EV. Error bars represent SD, and *n* = 8. * *P* < 0.05, ** *P* < 0.01, **** *P* < 0.001, parental EV vs. mutant EV, and mutant EV vs. complementation using unpaired *t*-test with Holm-Sidak correction.
(TIF)

S6 Fig (A) Percentage of stalled/collapsed forks during replication fork progression without stress from Fig 7A, and replication fork restart after replication stress from Fig 7B. Stalled/collapsed forks were considered any fiber stained with CldU only, and ‘other’ represents any DNA fiber that contained IdU staining. Numbers of fibers analyzed and replicates are as in Fig 7A and 7B. * *P* < 0.05 and ** *P* < 0.01, parental vs. mutant, and parental siCTRL vs. other siRNA treatments using Fisher’s exact test. (B) Influence of BRCA2 depletion on replication fork progression without stress and after stress, performed as in Fig 7A and 7B. Parental cells were treated with non-targeting siRNA (siCTRL) or with a pool of four BRCA2 siRNA (siBRCA2), as in Fig 7C. Numbers of fibers analyzed and statistics are as in Fig 7A and 7B.
(TIF)

S1 Table Sequences of sgRNAs and other oligonucleotides. (PDF)
