# Supplementary figures and images for: Distinct roles of RAD52 and POLQ in chromosomal break repair and replication stress response
Source: PLoS Genet. 2019 Aug 5;15(8):e1008319. doi: 10.1371/journal.pgen.1008319 (PMC6695211; doi:10.1371/journal.pgen.1008319)

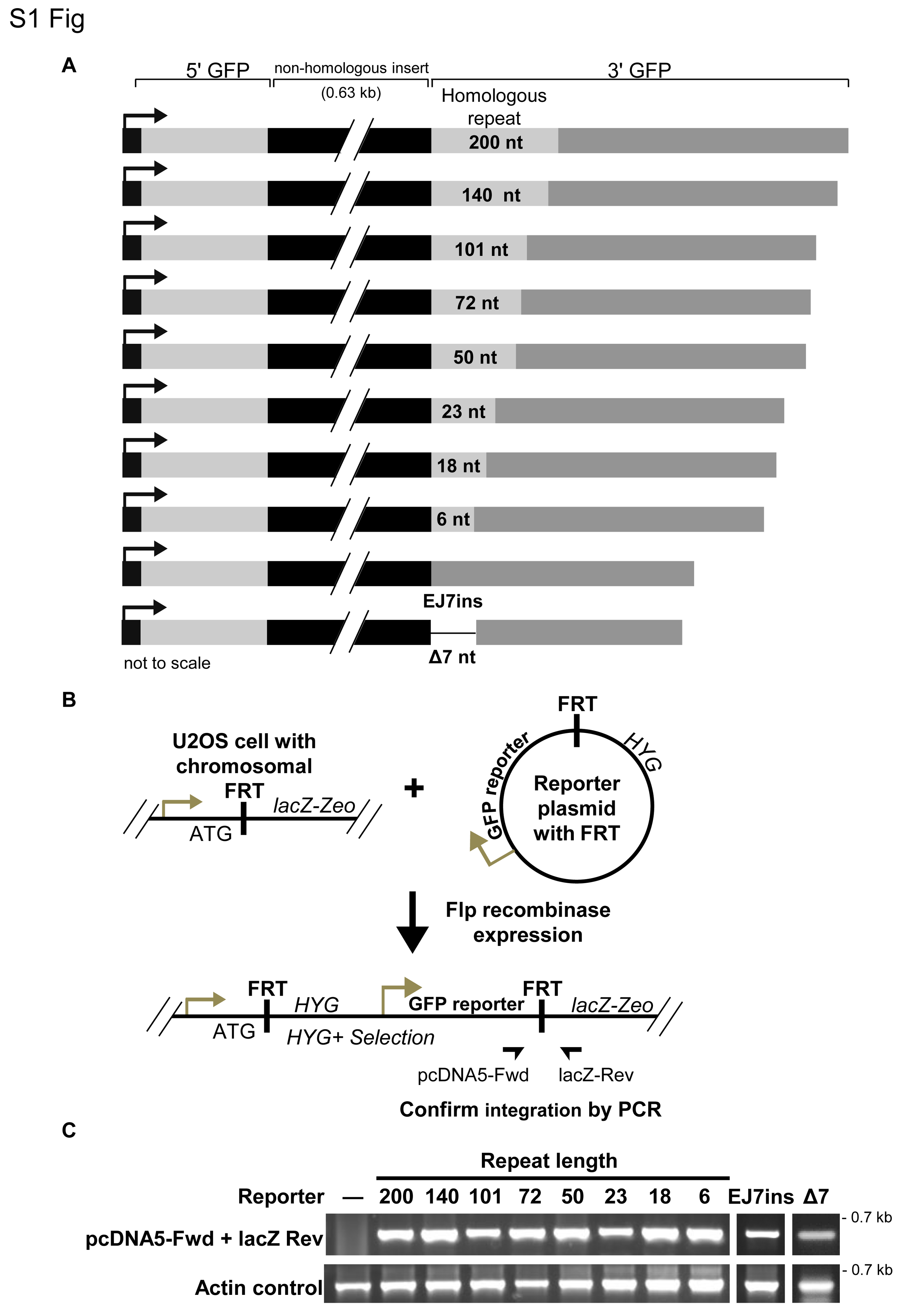

Supplement: S1 Fig — (A). Diagram of GFP reporter cassettes. (B). Schematic of the FRT/Flp system used to introduce the reporter cassettes into a specific chromosomal FRT locus in U2OS cells. (C) PCR products from parental (U2OS) cells with integrated reporters using primers that flank the downstream FRT site (pcDNA5-Fwd with lacZ-Rev). Parental cells without any integrated reporters were used as a negative control, and primers that amplify Actin were used as a positive control. (TIF) [file pgen.1008319.s001.tif]

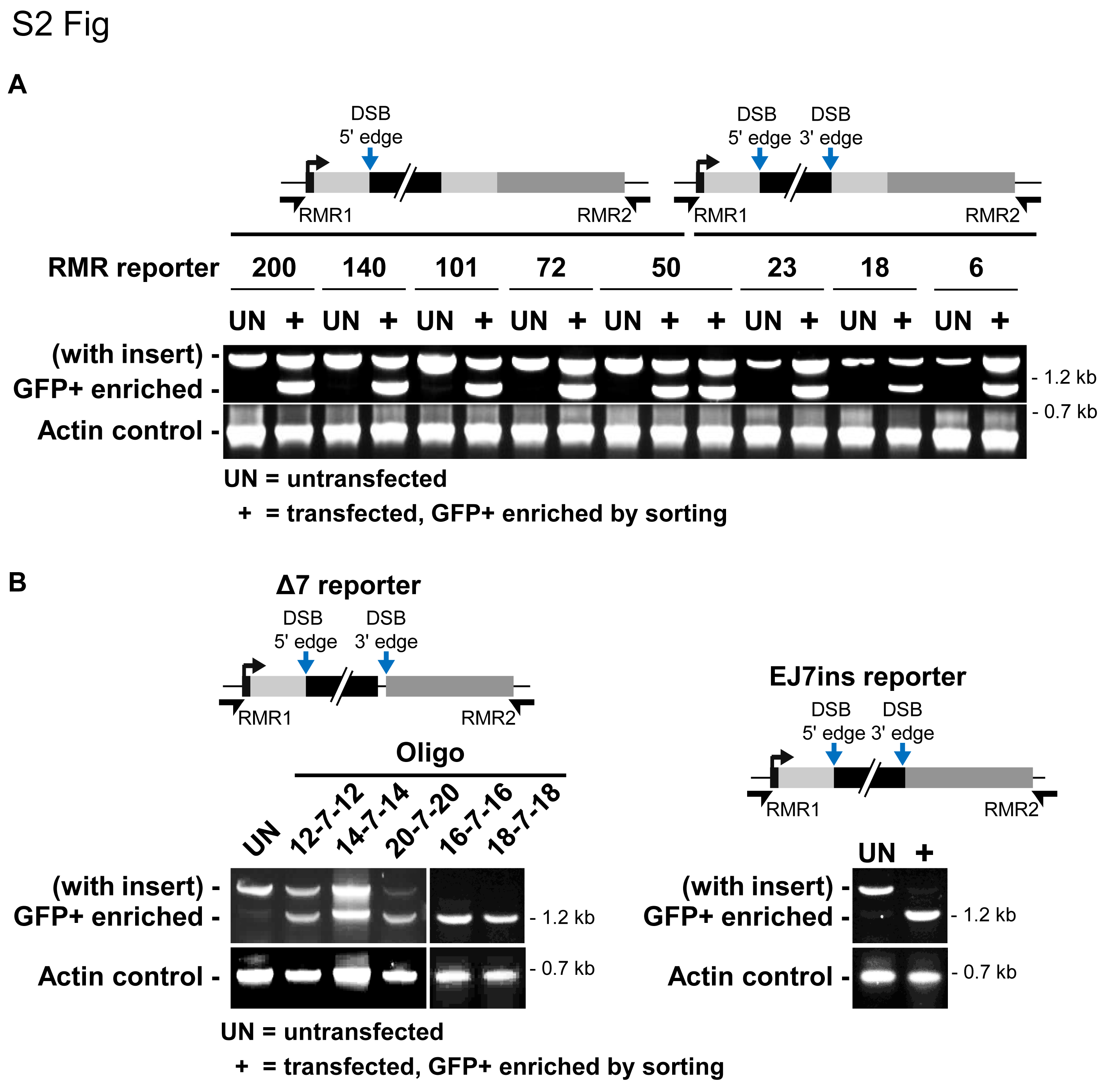

Supplement: S2 Fig — (A) PCR amplification products using primers that flank the GFP cassette (RMR1 and RMR2) for each reporter cell line after expressing the indicated sgRNA/Cas9 (5' edge and 5' & 3' edges), followed by cell sorting to enrich for GFP+ cells. (B) PCR amplification products using primers that flank the GFP cassette (RMR1 and RMR2) from the Δ7 reporter cassette with the indicated oligonucleotide and the EJ7ins reporter cassette, expressing the sgRNAs/Cas9 targeting the 5' & 3' edges of the non-homologous insert. UN, untransfected; +, GFP+ cells enriched by sorting. Primers that amplify Actin were used as a positive control. (TIF) [file pgen.1008319.s002.tif]

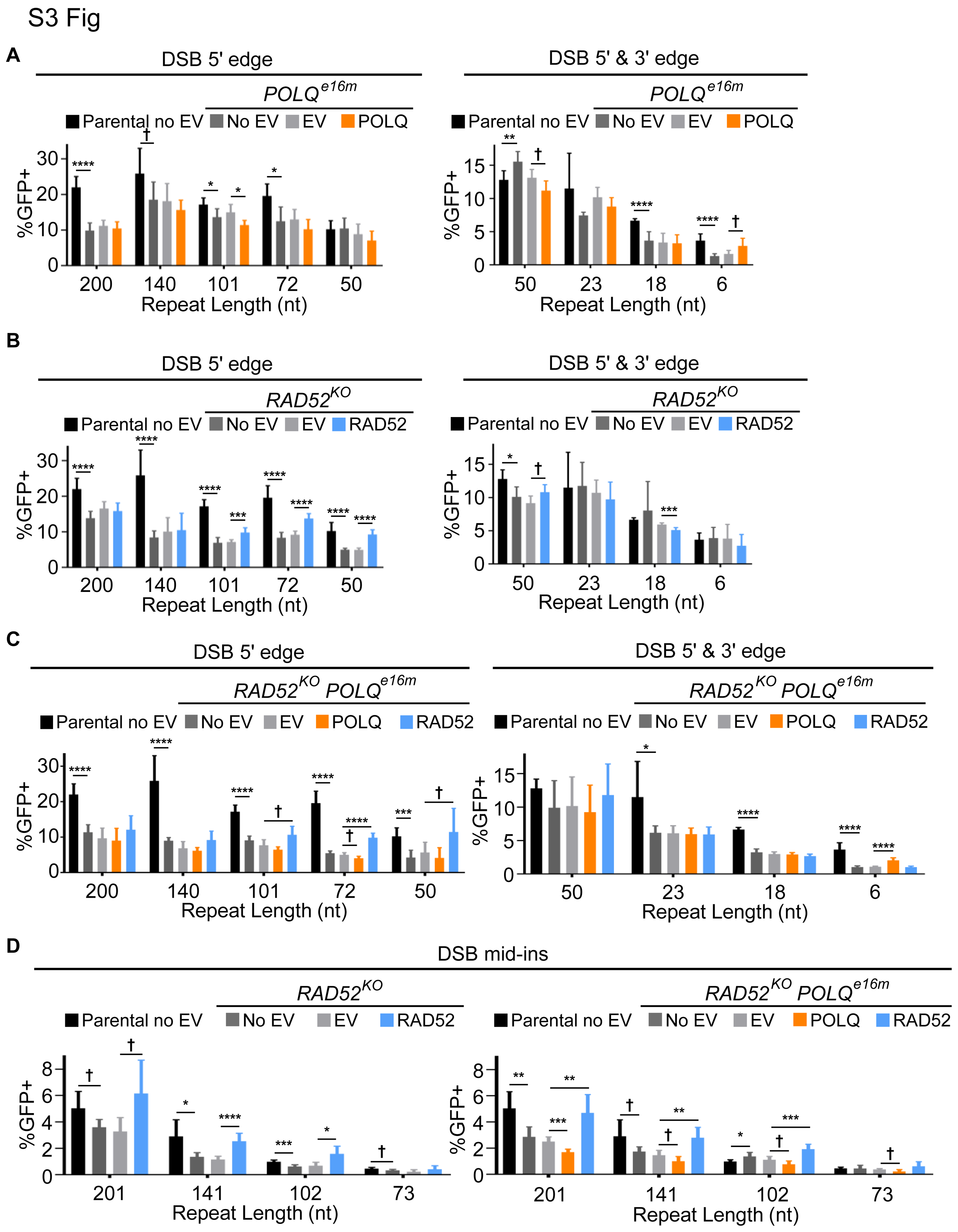

Supplement: S3 Fig — (A) Shown are frequencies for the parental and the POLQe16m cell line for RMR events induced with the 5’ edge DSB, and combination of 5’ and 3’ edge DSBs. Two independent clones were tested for each reporter in each cell line with four independent replicates for a total n = 8, except the parental 23 nt repeat where six independent clones were tested for n = 24. Error bars represent SD. * P < 0.05, ** P < 0.01, *** P < 0.005, **** P < 0.001, parental no EV vs. mutant (No EV), and mutant EV vs. complementation using unpaired t-test with Holm-Sidak correction. † P < 0.05 using unpaired t-test, but not significant when corrected for multiple comparisons (i.e., unadjusted P-value). (B) Shown are frequencies for the parental and the RAD52KO cell line for RMR events induced with the 5’ edge DSB, and combination of 5’ and 3’ edge DSBs. Experiments were performed as in panel (A), except for the RAD52KO 18 nt repeat where four independent clones were tested for n = 16. Statistics are as in (A). (C) Shown are frequencies for the parental and the RAD52KOPOLQe16m cell line for RMR events induced with the 5’ edge DSB, and combination of 5’ and 3’ edge DSBs. Experiments and statistics were performed as in (A). (D) Shown are frequencies for the parental, RAD52KO, and RAD52KOPOLQe16m cell lines for RMR events induced with the mid-ins DSB. Experiments and statistics were performed as in (A). (TIF) [file pgen.1008319.s003.tif]

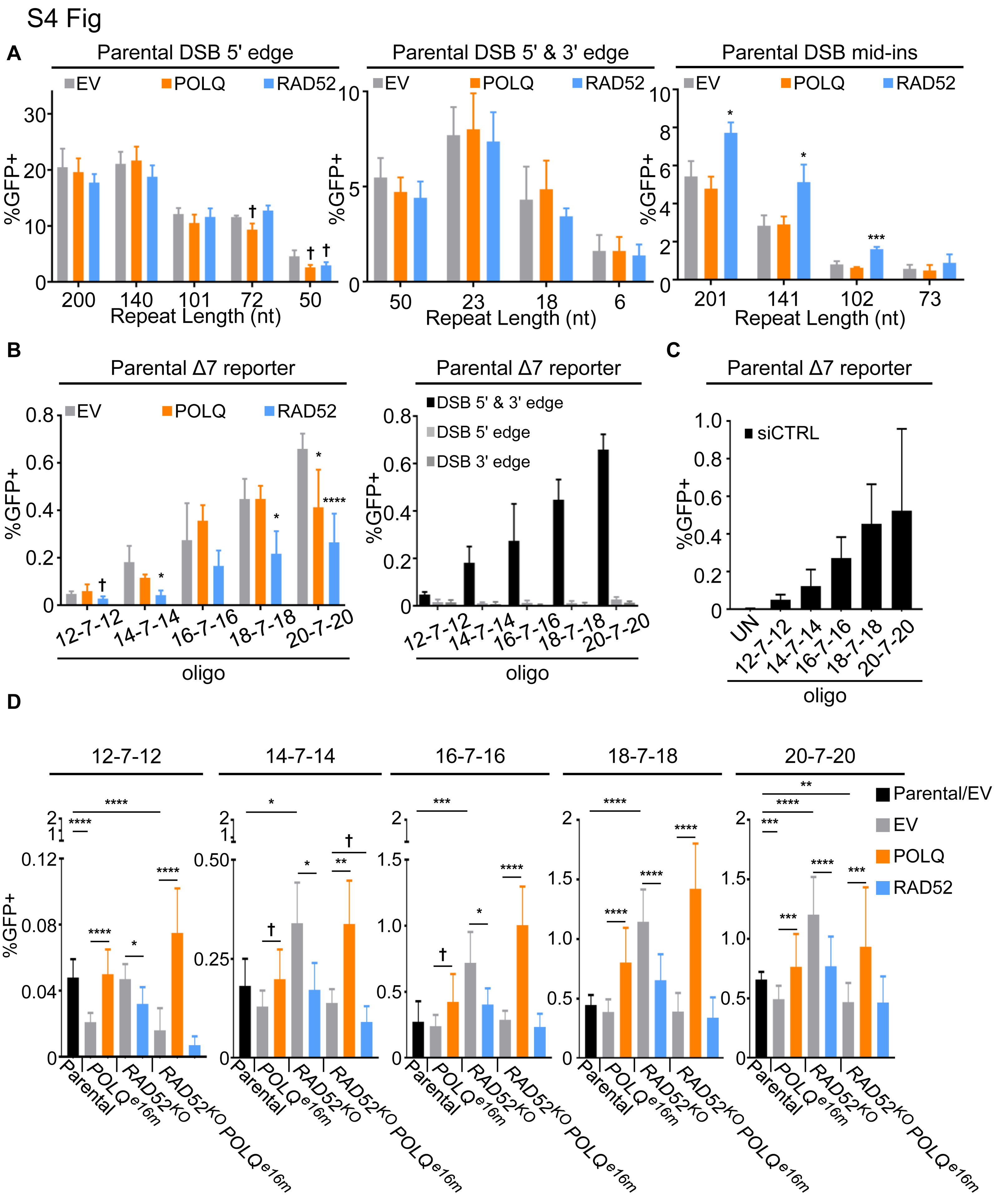

Supplement: S4 Fig — (A) Frequencies of RMR events from overexpression of POLQ and RAD52 in the parental cell line, normalized to transfection efficiency. The parental reporter cell lines were transfected with an expression vector for the sgRNA(s) and Cas9, as indicated, along with empty vector (EV), POLQ expression vector, or RAD52 expression vector. Error bars represent SD. Two independent clones were tested for each reporter with two independent replicates for a total n = 4. † P < 0.05 (unadjusted P-value), * P < 0.05, ** P < 0.01, EV vs. overexpression (POLQ or RAD52) using unpaired t-test with Holm-Sidak correction. (B) Overexpression of POLQ and RAD52 in the parental Δ7 reporter cell line. The cell lines were transfected with an expression vector for the sgRNA(s) and Cas9, and empty vector (EV), POLQ expression vector, or RAD52 expression vector, along with the 12-7-12, 14-7-14, 16-7-16, 18-7-18, or 20-7-20 oligonucleotides. Frequencies of GFP+ cells analyzed as in (A). Error bars represent SD. Two independent clones were tested with two replicates for a total n = 4, except parental EV where four replicates were analyzed for n = 8. † P < 0.05 (unadjusted P-value), * P < 0.05, **** P < 0.001, EV vs. overexpression using unpaired t-test with Holm-Sidak correction. Also shown are the percentages of GFP+ cells when targeting sgRNA(s) and Cas9 to the 5' edge, 3' edge, or 5' & 3' edge in the parental Δ7 reporter cell line with the 12-7-12, 14-7-14, 16-7-16, 18-7-18, or 20-7-20 oligonucleotides. Error bars represent SD. Two independent clones were tested with two independent replicates for a total n = 4, except DSB 5' & 3' edge where four independent replicates were analyzed for n = 8. (C) Percentages of GFP+ cells from the non-targeting siRNA (siCTRL) in Fig 5C (left panel) normalized to transfection efficiency including the 12-7-12, 14-7-14, 16-7-16, 18-7-18, and 20-7-20 oligonucleotides. UN, untransfected. Error bars represent SD. Two independent clones were tested with two replicates [file pgen.1008319.s004.tif]

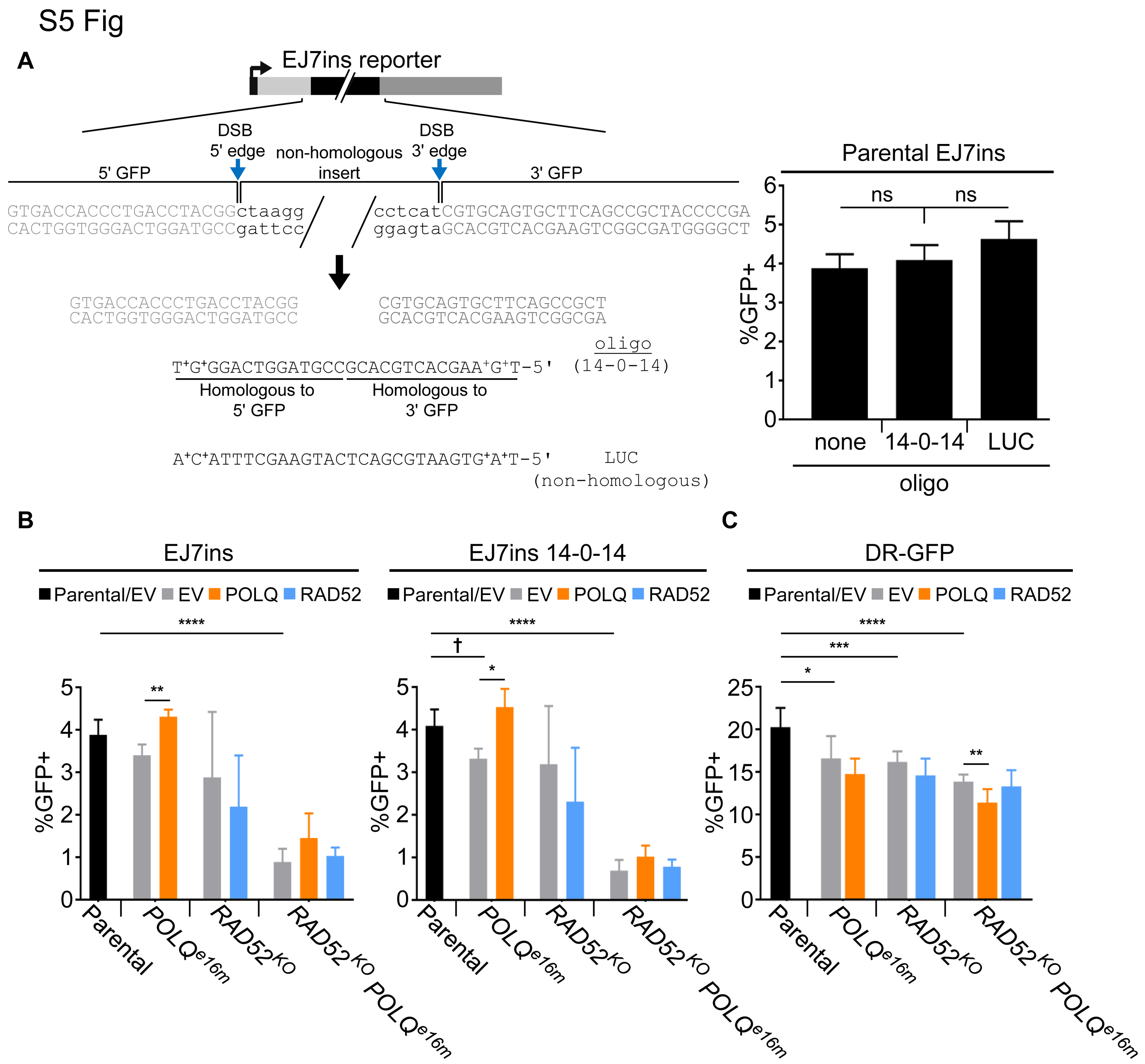

Supplement: S5 Fig — (A) Diagram of EJ7ins reporter in which targeted DSBs by sgRNAs and Cas9 to excise the non-homologous insert can restore GFP expression via EJ without indels. Shown are the percentages of GFP+ cells in the parental EJ7ins reporter cell line. Cells were transfected with expression vectors for the sgRNAs and Cas9, along with empty vector (EV), either in the absence (none) or presence of an oligonucleotide that contained 14 nt of homology to the 5' and 3' GFP sequences (14-0-14) or a non-homologous control oligonucleotide (LUC, 28 nt total sequence). The plus signs indicate phosphorothioate linkages. Error bars represent SD. Two independent clones were tested with two independent replicates for a total n = 4. ns, not significant, 14-0-14 vs. no oligonucleotide (none) and LUC-oligo using unpaired t-test with Holm-Sidak correction. (B) Percentages of GFP+ cells from Fig 6A complementation analysis, normalized to transfection efficiency but including parental EV. Number of cell lines tested and number of replicates as in (A). Error bars represent SD. † P < 0.05 (unadjusted P-value), * P < 0.05, ** P < 0.01, **** P < 0.001, parental EV vs. mutant EV, and mutant EV vs. complementation using unpaired t-test with Holm-Sidak correction. (C)Percentages of GFP+ cells from Fig 6B complementation analysis, normalized to transfection efficiency but including parental EV. Error bars represent SD, and n = 8. * P < 0.05, ** P < 0.01, **** P < 0.001, parental EV vs. mutant EV, and mutant EV vs. complementation using unpaired t-test with Holm-Sidak correction. (TIF) [file pgen.1008319.s005.tif]

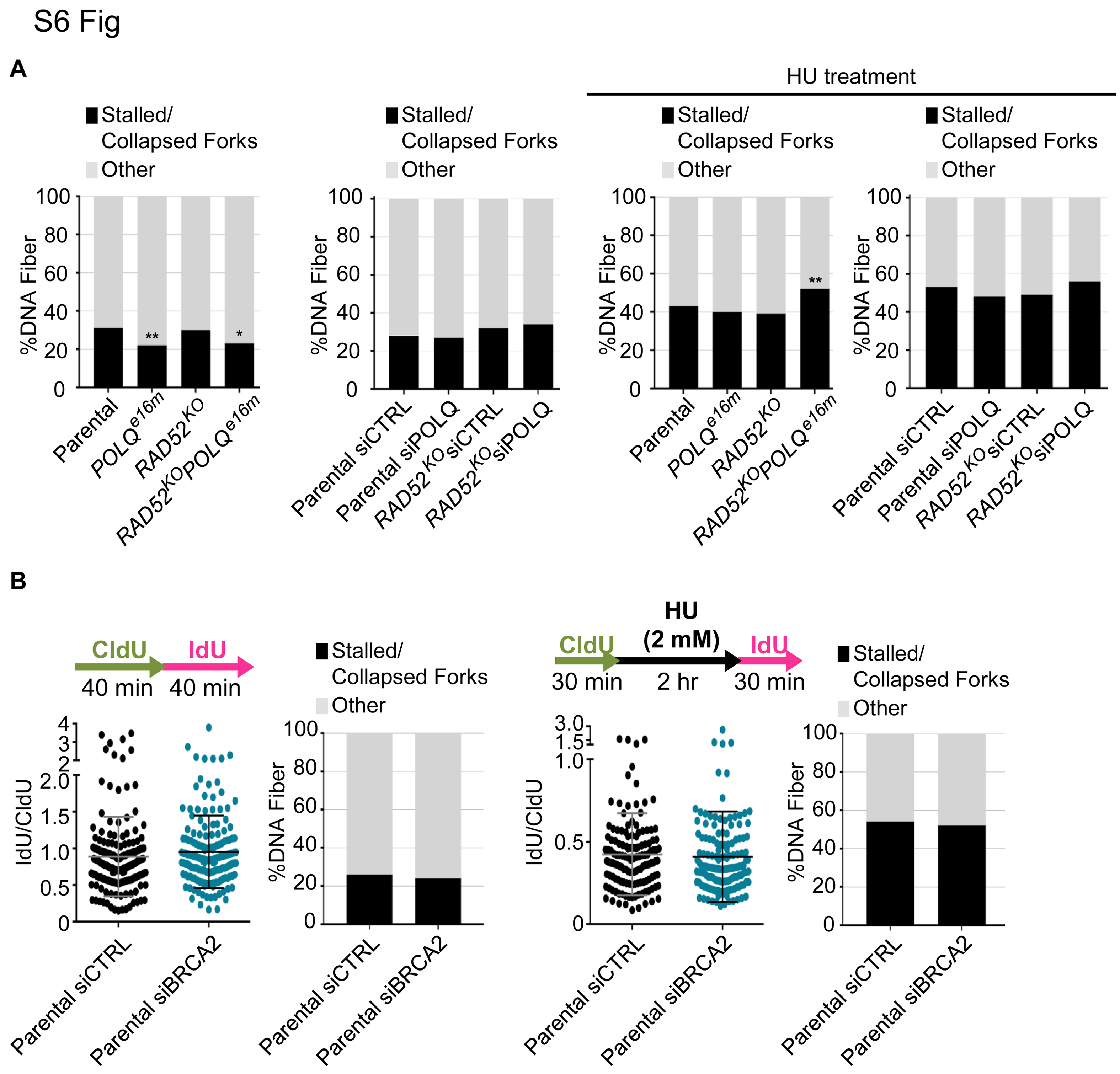

Supplement: S6 Fig — (A) Percentage of stalled/collapsed forks during replication fork progression without stress from Fig 7A, and replication fork restart after replication stress from Fig 7B. Stalled/collapsed forks were considered any fiber stained with CldU only, and ‘other’ represents any DNA fiber that contained IdU staining. Numbers of fibers analyzed and replicates are as in Fig 7A and 7B. * P < 0.05 and ** P < 0.01, parental vs. mutant, and parental siCTRL vs. other siRNA treatments using Fisher’s exact test. (B) Influence of BRCA2 depletion on replication fork progression without stress and after stress, performed as in Fig 7A and 7B. Parental cells were treated with non-targeting siRNA (siCTRL) or with a pool of four BRCA2 siRNA (siBRCA2), as in Fig 7C. Numbers of fibers analyzed and statistics are as in Fig 7A and 7B. (TIF) [file pgen.1008319.s006.tif]
