# Supplementary material for: Distinct roles of RAD52 and POLQ in chromosomal break repair and replication stress response
Source: PLoS Genet. 2019 Aug 5;15(8):e1008319. doi: 10.1371/journal.pgen.1008319 (PMC6695211; doi:10.1371/journal.pgen.1008319)
Supplement: S1 Table — (PDF) [file pgen.1008319.s007.pdf]

S1 Table. Oligonucleotide list.

Name	Purpose	Sequence (5' → 3'), *=phosphorothioate bond, siRNAs are pools of the 4 listed
POLQ ex 16 A	sgRNA	GTCGCAATATGCGAACTATC
POLQ ex 16 B	sgRNA	GACTTGTCAGGCCACTAGTG
RAD52 exon 3	sgRNA	GTACATAAGTAGCCGCATGGC
RAD52 exon 4	sgRNA	GTGCTACATTGAGGGTCATC
RAD52 exon 9	sgRNA	GCCGGAGCTTCCGCTGGTGCG
RMR Reporter 5' edge DSB	sgRNA	ACCACCCTGACCTACGGCTA
RMR50 Reporter 3' edge DSB	sgRNA	GGCACGGGCAGCTTGCCATG
RMR23 Reporter 3' edge DSB	sgRNA	GTCAGGGTGGTCACGAGATG
RMR18 Reporter 3' edge DSB	sgRNA	CGTAGGTCAGGGTGGTCATG
RMR6 Reporter 3' edge DSB	sgRNA	AGCACTGCACGCCGTAGATG
RMRΔ7 Reporter 3' edge	sgRNA	GGGTAGCGGCTGAAGCAATG
Middle non-homologous insert (mid-ins)	sgRNA	GCCGCGCAGCAACAGATGG
DR-GFP	sgRNA	GGATAACAGGGTAATACCTA
12-7-12	oligonucleotide	G*C*GGCTGAAGCACTGCACGCCGTAGGTCA*G*G
14-7-14	oligonucleotide	T*A*GCGGCTGAAGCACTGCACGCCGTAGGTCAGG*G*T
16-7-16	oligonucleotide	G*G*TAGCGGCTGAAGCACTGCACGCCGTAGGTCAGGGT*G*G
18-7-18	oligonucleotide	G*G*GGTAGCGGCTGAAGCACTGCACGCCGTAGGTCAGGGTGG*T*C
20-7-20	oligonucleotide	T*C*GGGGTAGCGGCTGAAGCACTGCACGCCGTAGGTCAGGGTGGTC*A*C
14-0-14	oligonucleotide	T*G*AAGCACTGCACGCCGTAGGTCAGG*G*T
LUC-oligo	oligonucleotide	A*C*ATTTCGAAGTACTCAGCGTAAGTG*A*T
PQ1	primer	CACCGACTTGTCAGGCCACTAGTG
PQ2	primer	CAGCCCCCTGAAAGACTCTG
PQ3	primer	AAACATGCTCCAGCTGATGGAAGT
pcDNA5 forward	primer	ATTGCATCGCATTGTCTGAG
lacZ reverse	primer	GTTTTCCCAGTCACGACGTT
Actin forward	primer	ACTGGGACGACATGGAGAAG
Actin reverse	primer	AGGAAGGAAGGCTGGAAGAG
RMR1	primer	CGCAAATGGGCGGTAGGCGTG
RMR2	primer	TAGAAGGCACAGTCGAGG
RMR140	primer	ACGACCCCCTAGGCCTCATGACGTAAACGGCCACAAGTT
RMR101	primer	ACGACCCCCTAGGCCTCATGAGGGCGATGCCACCTAC
RMR72	primer	ACGACCCCCTAGGCCTCATCCTGAAGTTCATCTGCACCA
RMR50	primer	ACGACCCCCTAGGCCTCATGGCAAGCTGCCCGTGCCC
RMR23	primer	ACGACCCCCTAGGCCTCATCTCGTGACCACCCTGACCTA
RMR18	primer	ACGACCCCCTAGGCCTCATGACCACCCTGACCTACGG
RMR6	primer	ACGACCCCCTAGGCCTCATCTACGGCGTGCAGTGCTT
Δ7 reporter	primer	ACGACCCCCTAGGCCTCATTGCTTCAGCCGCTACCCCGA
RMR reverse	primer	GCGATGCAATTTCCTCATTT
Actin RTPCR forward	primer	GGAAATCGTGCGTGACATTA
Actin RTPCR reverse	primer	AGGAAGGAAGGCTGGAAGAG
POLQ RTPCR forward	primer	CTGACCTGCAAAGAGCAATG
POLQ RTPCR reverse	primer	ACCCCTCTTCAACTCCCACT
non-targeting siRNA (siCTRL)	siRNA target sequence	UGGUUUACAUGUCGACUAA
RAD52 (#1)	siRNA target sequence	GGAAAUAUGAUCCAUCUUA
RAD52 (#2)	siRNA target sequence	UAAUUAAUCUGGCCAAUGA
RAD52 (#3)	siRNA target sequence	CAGAAGGUGUGCUACAUUG
RAD52 (#4)	siRNA target sequence	GAUGUUGGUUAUGGUGUUA
POLQ (#1)	siRNA target sequence	CAACAACCCUUAUCGUAAA
POLQ (#2)	siRNA target sequence	GGACUACUAUUGAUUGGUA
POLQ (#3)	siRNA target sequence	CGACUAAGAUAGAUCAUUU
POLQ (#4)	siRNA target sequence	AAACGGGCCUCUUUAGAUA
BRCA2 (#1)	siRNA target sequence	GAAACGGACUUGCUAUUUA
BRCA2 (#2)	siRNA target sequence	GUAAAGAAAUGCAGAAUUC
BRCA2 (#3)	siRNA target sequence	GGUAUCAGAUGCUUCAUUA
BRCA2 (#4)	siRNA target sequence	GAAGAAUGCAGGUUUAAUA
